# Understanding the substrate recognition and catalytic mechanism of 2-O-methyl fucosidases from glycoside hydrolase family 139

**DOI:** 10.1016/j.jbc.2025.110407

**Published:** 2025-06-20

**Authors:** Zak McIver, Alicia Moraleda-Montoya, Zongjia Chen, Ruwan Epa, David Starns, Matthew Davy, Mikel García-Alija, Arnaud Basle, Mario Schubert, Didier Ndeh, Beatriz Trastoy, Spencer J. Williams, Marcelo E. Guerin, Alan Cartmell

**Affiliations:** 1 Department of Biology, University of York, York, United Kingdom; 2 Department of Chemistry, York Structural Biology Laboratory, York, United Kingdom; 3 York Biomedical Research Institute, University of York, York, United Kingdom; 4 Structural Glycoimmunology Laboratory, Biobizkaia Health Research Institute, Barakaldo, Spain; 5 School of Chemistry and Bio21, Molecular Science and Biotechnology Institute, University of Melbourne, Parkville, Victoria, Australia; 6 Faculty of Biological Sciences, School of Molecular and Cellular Biology, University of Leeds, Leeds, United Kingdom; 7 Department of Chemistry, University of York, York, United Kingdom; 8 Newcastle University Biosciences Institute, Medical School, Newcastle University, Newcastle upon Tyne, United Kingdom; 9 FU Berlin, Department of Biology, Chemistry and Pharmacy, Berlin, Germany; 10 The James Hutton Institute, School of Life Sciences, University of Dundee, Dundee, United Kingdom; 11 Ikerbasque, Basque Foundation for Science, Bilbao, Spain; 12 Structural Glycobiology Laboratory, Department of Structural and Molecular Biology, Molecular Biology Institute of Barcelona (IBMB), Spanish National Research Council (CSIC), Barcelona, Catalonia, Spain

**Keywords:** glycoside hydrolase, plant cell wall, complex glycan, enzyme mechanism, enzyme structure

## Abstract

Rhamnogalacturonan II is one of the most complex plant cell wall carbohydrates and is composed of 13 different sugars and 21 different glycosidic linkages. It is abundant in fruit and indulgence foods, such as chocolate and wine, making it common in the human diet. The human colonic commensal *Bacteroides thetaiotaomicron* expresses a consortium of 22 enzymes to metabolize rhamnogalacturonan II, some of which exclusively target sugars unique to rhamnogalacturonan II. Several of these enzyme families remain poorly described, and, consequently, our knowledge of rhamnogalacturonan II metabolism is limited. Chief among the poorly understood activities is glycoside hydrolase (GH) family 139, which targets **α**1,2-2*O*-methyl L-fucoside linkages, a sugar residue not found in any other plant cell wall complex glycans. Although the founding enzyme BT0984 was placed in the RG-II degradative pathway, no GH139 structure or catalytic blueprint had been available. We report the crystal structures of BT0984 and a second homolog revealing that the family operates with inverting stereochemistry. Using these data, we undertook a mutagenic strategy, backed by molecular dynamics, to identify the important substrate binding and catalytic residues, mapping these residues throughout the GH139 family revealing the importance of the *O*2 methyl interaction of the substrate. We propose a catalytic mechanism that uses a non-canonical Asn as a catalytic base and shares similarity with L-fucosidases/L-galactosidases of family GH95.

The plant cell wall is a woven matrix of complex glycans and is the dominant source of organic carbon in the terrestrial biosphere (1, 2). Bacterial and fungal degradation of the plant cell wall is critical to making this organic carbon available to other organisms as part of the carbon cycle (3). The degradation of this rich organic carbon sink is a difficult undertaking due to the number of different plant cell wall glycans and the varied properties they possess (4).

An example of these complex glycans is pectins, which are chemically diverse and abundant in primary cell walls (5, 6). Among them, rhamnogalacturonan II (RG-II) stands out as the most structurally intricate and is widely regarded as one of the most complex glycans found in nature. RG-II is notably abundant in fruit and luxury goods such as chocolate and wine, contributing to its presence in the human diet (6, 7, 8). This complex glycan is composed of up to 13 distinct monosaccharides linked by 21 different glycosidic linkages, some of which are unique to RG-II (6) (Fig. 1*A*).

**Figure 1:**
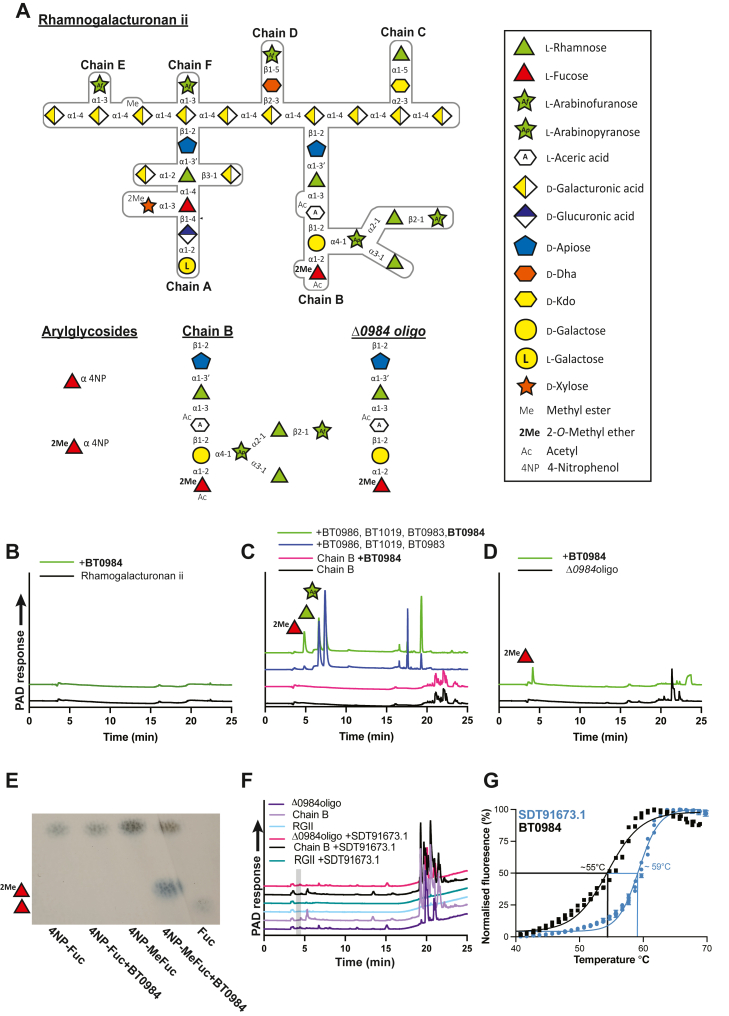
**GH139 enzymes cleave RG-II glycans: substrate structures, activity and thermal stability**. *A*, structure of rhamnogalacturonan ii and fragment glycan substrates used in this study; (*B*) *high*-performance anion exchange chromatography (HPAEC) of BT0984^GH139^ against rhamnogalacturonan ii; (*C*) HPAEC of BT0984^GH139^ against isolated chain B; (*D*) HPAEC of BT0984^GH139^ against *Δ0984oligo*; (*E*) thin layer chromatography of BT0984^GH139^*versus* 4NP-α-L-fucopyranoside and 4NP-α-L-2*O*-methylfucopyranoside; (*F*) HPAEC of SDT91673.1^GH139^*versus* rhamnogalacturonan ii, isolated chain B, and *Δ0984oligo*; the *grey box* indicates the expected position of 2*O*-methylfucose and shows it is absent. Enzyme concentrations used were 1 μM in 10 mM MOPS buffer pH 7.0 with 150 mM NaCl. *G,* DSF analysis of GH139 stability.

The structure of RG-II features an α1,4 poly-D-galacturonic (PG) acid backbone, which can be decorated with up to six side chains (A-F) (6). Chains E and F are single L-arbinofuranose (Araf) residues linked *via* α1,3 bonds; however, chain F lies opposite chain A, while chain E has been identified in RG-II extracted from wine (8) but not RG-II from apple (6). Chains C and D are disaccharides attached to the PG backbone through α2,3 and β2,3 linkages, respectively, involving the acidic sugars 2-keto-3-deoxy-D-lyxo-heptulosaric acid (Dha) and 3-deoxy-D-manno-oct-2-ulosonic acid (*Kdo*). Chain D is terminated by β1,5-Araf, while chain C ends with α1,5-L-rhamnose (Rha) (6, 8). Chains A and B are longer oligosaccharides—an octasaccharide and a nonasaccharide, respectively—that contribute to the complexity of RG-II. Both chains are linked to the backbone by a β1,2-linked disaccharide of D-apiose α1,3-linked to Rha (Fig. 1*A*). The apiose of chain A, but not B, allows the formation of dimers through borate cross-linking. This borate-mediated dimerization is crucial for cell wall stability, as the loss of these cross-links under boron deficiency compromises the integrity of the cell wall matrix, underscoring the vital role of RG-II in plant cell wall physiology (9).

Due to the structural complexity of RG-II, it was initially unclear whether a single bacterial species could metabolize it. However, in 2017 a landmark study demonstrated that the human gut bacterium *Bacteroides thetaiotaomicron* VPI-5482 (*B. theta*) is capable of degrading RG-II. Analysis of the *B. theta* RG-II degradome revealed it can break down all but one glycosidic linkage in RG-II(6). The genes encoding this ability are distributed across three polysaccharide localization loci (PULs) (6), which are co-localized clusters of genes that are co-regulated in response to a particular glycan (10). These PULs are not only conserved in other *Bacteroides* spp. from the human gut microbiota (HGM) but are also found in organisms from broader environmental niches. The PUL genes encode carbohydrate active enzymes (CAZymes), primarily glycoside hydrolases (GH), which are classified into sequence-based families in the Carbohydrate active enzyme database (CAZy) (11). These families can then be grouped into clans based on shared folds, mechanisms, and evolutionary origins.

The initial characterization of the *B. theta* RG-II degradome identified over 20 CAZymes, several of which exhibited novel catalytic functions and led to the establishment of new GH families (6, 12). However, some of the foundational enzymes of these new families remain to be fully characterized, such as those in family GH139, which targets the α1,2-linked 2O-methyl-L-fucose (MeFuc) in chain B of RG-II, which is required for complete metabolism of RG-II.

The *B. theta* protein BT0984 was the founding member of GH139 (BT0984^GH139^) and is specific for the α1,2-linked MeFuc in chain B(6). While L-fucose is a common component of both plant (13) and mammalian glycans (14), where it is always α-linked, MeFuc is much rarer (6, 15). Existing fucosidases belong to families GH29 (16), GH95 (17), GH141 (6), GH151 (18), and GH168 (19); however, these only target L-fucose, and not MeFuc. To date, family GH139 is the only family known to be able to degrade α1,2-linked MeFuc, making it essential for RG-II metabolism and a feature of RG-II-degrading bacteria. Despite its pivotal role in RG-II metabolism, family GH139 remains structurally and mechanistically uncharacterized. The key determinants of its specificity, as well as its catalytic residues and mechanism, are yet to be defined.

In this report, we present the 3D structure of BT0984^GH139^, alongside its homolog SDT91673.1^GH139^ from *Verrucomicrobium* sp. GAS474, an organism isolated from forest soil. By combining structural insights with enzymatic data on defined substrates, we elucidate the catalytic mechanism and identify the key residues required for binding and catalysis at the −1/+1 subsites. Our findings reveal mechanistic similarities with the GH95 family, including the proposal of a non-canonical amino acid as the catalytic base. However, notable differences in the positioning of the amino acid residues in the −1 subsite (the site of catalysis) suggest a distinct binding mode within family GH139, highlighting its unique adaptation for MeFuc degradation.

## Results

### BT0984^GH139^ is an *exo*-acting 2-*O*-methyl-**α**-L-fucosidase

BT0984^GH139^ specifically cleaves α1,2 linked MeFuc in partially digested RG-II but cannot access this residue within the intact polysaccharide (6) (Fig. 1*B*). However, once the preceding arabinofuranose (Ara*f*) and rhamnose (Rha) residues have been removed, BT0984^GH139^ can efficiently remove MeFuc from isolated chain B (Fig. 1*C*). It can also cleave MeFuc from “Δ*0984* oligo,” which terminates in MeFuc at the non-reducing end (Fig. 1*D*). This oligosaccharide was generated from growing a Δ*bt0984* mutant of *B. theta* on RG-II and is the same structure as chain B once the Ara*f* and Rha residues are removed (Fig. 1). BT0984^GH139^ exhibited no activity on 4-nitrophenyl α-L-fucoside (4NP-Fuc) but displayed activity on 4-nitrophenyl 2-*O*-methyl-α-L-fucoside (4NP-MeFuc) (Fig. 1*E*). This demonstrates that the 2*O* methyl substituent on fucose is essential for activity on aryl substrates. A large increase in activity was observed on the Δ*0984* oligo, indicating that recognition of the reducing-end sugars by the enzyme is required for optimal activity. With RG-II being ubiquitous in primary plant cell walls it is also a common component in soil microbiomes. To test whether an ortholog of BT0984^GH139^ from a soil environment was also an *exo*-acting 2-*O*-methyl-α-L-fucosidase, we cloned and recombinantly expressed, in *Escherichia coli*, SDT91673.1^GH139^ from *Verrucomicrobium* sp. GAS474; an organism isolated from a forest in Harvard, USA. However, SDT91673.1^GH139^ showed no detectable activity on any of the substrates tested (Fig. 1*F*). Thermal melt analysis of BT0984^GH139^ revealed a melting temperature (Tm) of ∼48 °C, whilst SDT91673.1^GH139^ showed a Tm of ∼59 °C, indicating it is more thermostable protein than BT0984^GH139^ (Fig. 1*G* and Table 1).

**Table 1:** Specific activities of BT0984GH139 and it mutants

Enzyme	Specific activity (min^−1^)	Relative activity (%)
WT	(2.95 ± 0.12) x 10^3^	100
W162A	(1.22 ± 0.10) x 10^2^	4
W403A	(2.33 ± 0.16) x 10^−1^	0.01
Q411A	(3.99 ± 0.27) x 10^2^	13.5
N412A	NQ	NQ
E472A	(4.41 ± 0.05) x 10^1^	0.14
E472Q	NQ	NQ
W490A	(3.26 ± 0.40) x 10^1^	0.11
E561A	NQ	NQ
E561Q	NQ	NQ
N639A	(3.07 ± 0.01) x 10^2^	10.4
E641A	(6.73 ± 0.12) x 10^1^	0.2
E641Q	(1.17 ± 0.01) x 10^1^	0.4
W683A	(5.76 ± 0.53) x 10^−1^	0.01
Q411A/N412A	(1.17 ± 0.01) x 10^0^	0.03
Q411A/E561Q	NQ	NQ
N412A/E561Q	NQ	NQ
Q411A/N412A/E561Q	NQ	NQ

Enzymes were assayed against 0.1 mM Δ0984oligo at 37 °C in 10 mM MOPS pH 7.0 with 150 mM NaCl. Assays were performed in triplicate and errors are the standard error of the mean.

### BT0984^GH139^ cleaves substrate with inversion of anomeric stereochemistry

**Figure 2:**
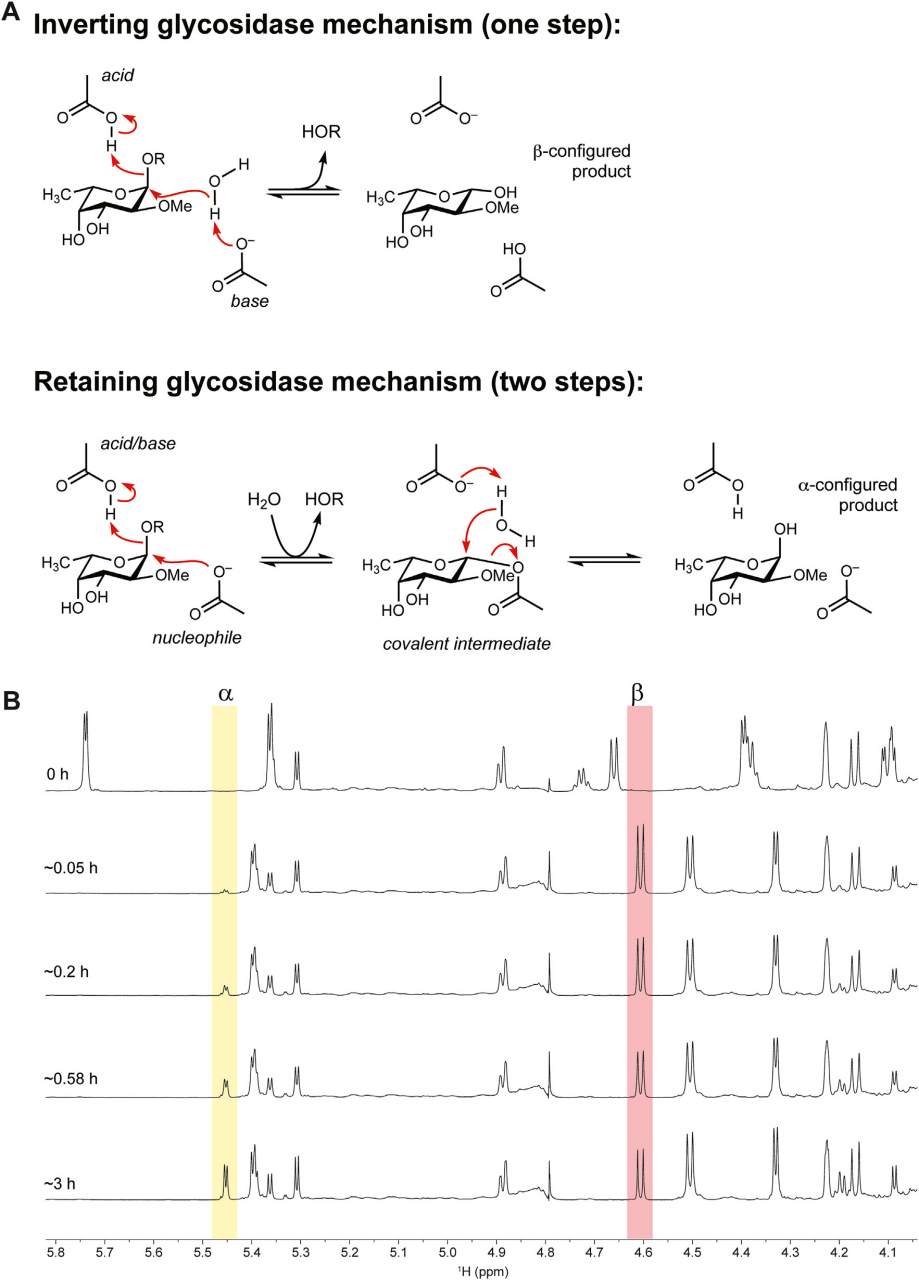
**BT0984^GH139^ cleaves 2*O*-methyl-α-fucoside linkages with inversion of stereochemistry**. *A*, the two most common catalytic mechanisms employed by glycoside hydrolases. *B*, NMR time course of BT0984^GH139^ cleavage of *Δbt0984* oligo in D_2_O. Enzyme concentration and substrate amounts used were 50 μM and 0.5 mg in 10 mM HEPES buffer pH 7.0 with 150 mM NaCl.

As the founding member of the GH139 family, we sought to determine the mechanism by which BT0984^GH139^ cleaves the glycosidic bond. Therefore, we employed ^1^H NMR spectroscopy to monitor the anomeric configuration of the MeFuc product immediately after hydrolysis. The anomeric configuration of sugar refers to the stereochemistry of the C1 hydroxyl, which can be α or β. In L-fucose adopting a ^1^*C*_4_ conformation, the α configuration places the C1 hydroxyl in the axial position, perpendicular to the plane of the ring, while the β configuration places it equatorial and roughly in the plane of the sugar ring. Although several mechanisms exist by which GHs can cleave glycosidic bonds most follow one of two well-characterized mechanisms (20): an inverting mechanism that involves a single-step displacement reaction that inverts the anomeric configuration of the sugar residue in the −1 subsite, or a retaining mechanism that involves a two-step, double displacement mechanism that proceeds *via* a covalent glycosyl-enzyme intermediate and ultimately retains the anomeric configuration of the −1 sugar (Fig. 2*A*). Both mechanisms typically require a pair of carboxylate acid residues (Asp or Glu) that serve distinct catalytic roles. In the inverting mechanism, they act as a general base and a general acid, whereas in the retaining mechanism, they act as a nucleophile and a general acid/base (Fig. 2*A*).

To investigate the BT0984^GH139^ mechanism, we initially used the synthetic substrate 4NP-MeFuc and monitored the appearance of anomeric signals of the product. ^1^H NMR spectra were acquired before and after the addition of BT0984^GH139^, with monitoring over a 24h time course. The anomeric configuration of MeFuc produced from 4NP-MeFuc is dictated by the mechanism of BT0984^GH139^, but over time will reach an equilibrium between the α and β forms, requiring careful timing to establish the initial configuration. Unfortunately, despite using enzyme concentrations in excess of 80 μM, the rate at which BT0984^GH139^ produced MeFuc was slower than the rate of mutarotation, preventing clear identification of the stereochemical outcome.

We repeated the above experiment using the *Δbt0984* oligo, which BT0984^GH139^ hydrolyzes much more quickly than 4NP-MeFuc. Immediately upon addition BT0984^GH139^, a marked increase in the signal of the β anomeric signal of MeFuc at δ 4.6 ppm was observed, while the α-anomer signal at δ 5.45 ppm remained negligible until approximately 0.2 h after enzyme addition (Fig. 2*B*). By 3 h the anomeric signals had reached equilibrium. These data indicate BT0984^GH139^ produces β-MeFuc and performs cleavage with inversion of the anomeric configuration of the α-MeFuc linkage. Full ^1^H 1D assignments of the *Δbt0984* oligo before and after incubation are shown in Fig. S1 and Tables S1 and S2. Fig. S2 displays ^1^H-^13^ C HSQC and ^1^H-^13^ C HMQC 2D spectra for the released MeFuc compared to a MeFuc standard.

### The tertiary structure of GH139 enzymes is similar to GH95

**Figure 3:**
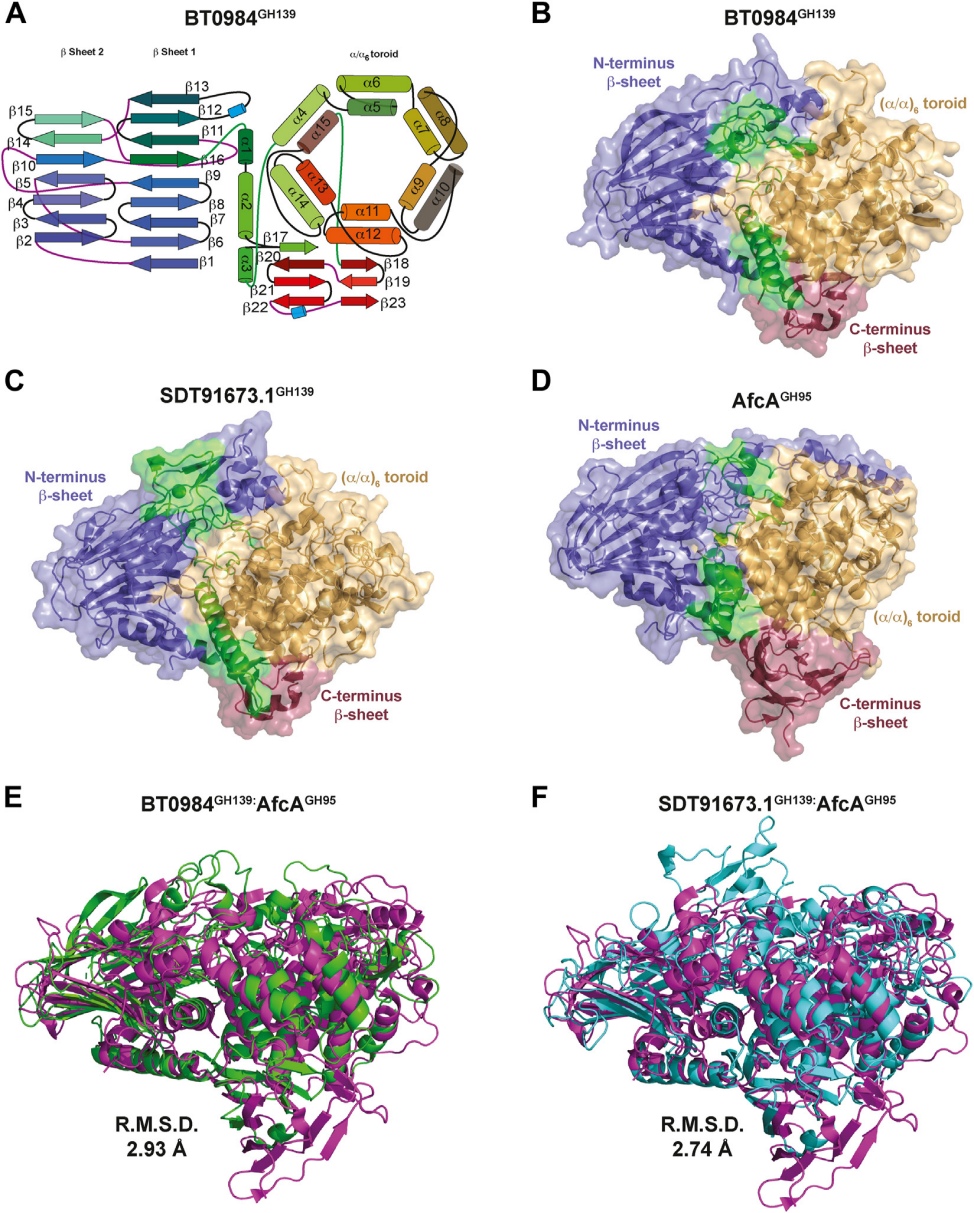
**GH139 enzymes share a toroidal (α/α)_6_ structure with Afca^GH95^**. *A*, *cartoon* schematic showing the general 3-D structure of GH139 enzymes. *B, C, D*, transparent surface views of the three enzymes with ribbon diagram of protein structures with the N-terminal domain coloured *slate blue*, the (α/α)_6_*toroid light orange*, and the C-terminal *raspberry red*, with interdomain linkers shown in *green*: (B) BT0984^GH139^, (*C*) SDT91673.1^GH139^, and (*D*) AfcA^GH95^. *E*, structural overlay of BT0984^GH139^ (*green*) and AfcA^GH95^ (*magenta*). *F*, structural overlay of SDT91673.1^GH139^ (*cyan*) and Afca^GH95^ (*magenta*).

Crystals of BT0984^GH139^ formed in space group P2_1_2_1_2_1_ and diffracted to a resolution of 2.7 Å, while crystals of SDT91673.1^GH139^ formed in space group P 6_5_22 and diffracted to 2.05 Å, providing good quality electron density maps in both cases (Fig. S3 and Table S3). Both proteins adopt a three-domain architecture, consisting of an N-terminal β-sheet domain leading into an α-helical domain composed of a core (α/α)_6_ toroid structure. This is followed by a smaller C-terminal domain composed of two anti-parallel β-sheets, with the upper and lower sheets containing three and four strands, respectively (Fig. 3, *A* and *B, C*).

The N-terminal domains of BT0984^GH139^ and SDT91673.1^GH139^ are composed of two anti-parallel β-sheets: the bottom sheet contains nine strands, while the top sheet contains six. An extended loop between β-strands 12 and 13 interacts with the (α/α)_6_ toroid domain, which is longer in SDT91673.1^GH139^. Three α-helices then lead from β-strand 16, the first two wrap around the opposite side of the (α/α)_6_ toroid, while the third sits in between the two domains. A small β-strand is between helix α2 and three forms the first strand of the C-terminal β-sheet domain (Fig. 3, *A*–*C*).

The (α/α)_6_ toroid domain is composed of six inner helices circled by 6 outer helices. The first outer helix, α4, leads into the inner helix α5, which pairs with outer helix α6. The helices alternate consecutively from the inner to outer rings, forming the toroid structure until α15 becomes the final inner helix running parallel to α4 and completing the (α/α)_6_ toroid fold (Fig. 3*A*). Following the toroid domain is a final anti-parallel β-sheet domain composed of two β-sheets. The smaller bottom sheet contains strands β18 (connecting directly to the (α/α)_6_ toroid), β19 and β23, marking the end of the fold, whilst the larger bottom sheet comprises the strands β17 (contributed from the loop between helix α2 and 3), β20, β21, and β22 (Fig. 3*A*).

A structural comparison search using Foldseek Search (21) returned GH95 as the most closely related GH family in terms of structure, while sequence identities were 12% to 15%. The best characterized of these matches, both structurally and biochemically, is the GH95 AfcA (PDB: 2EAE) from *Bifidobacterium bifidum*. AfcA^GH95^ comprises the same three domains in the same order as BT0984^GH139^ and SDT91673.1^GH139^ (Fig. 3, D–F). Some differences in the β-sheets exist as although the AfcA^GH95^ N-terminal domain also consists of 15 strands it has seven in the top sheet and eight in the bottom, as opposed to six and nine in BT0984^GH139^ and SDT91673.1^GH139^. The extended loop of the N-terminal domain, equivalent to the loop between β-strands 12 and 13 in BT0984^GH139^ and SDT91673.1^GH139^, is significantly extended in AfcA^GH95^ and wraps around the (α/α)_6_ toroid domain (Fig. 3*D*). The C-terminal domain has an additional 4 β-strands, two in the top and two in the bottom sheet. The (α/α)_6_ toroid has no significant structural differences. Pairwise structural alignment, using the JCE algorithm *via* the RCSB PDB interface (22), of BT0984^GH139^ with SDT91673.1^GH139^ and AfcA^GH95^ gave RMSD values of 1.74 Å and 2.93 Å, respectively. Comparison of the individual domains gives RMSD values of 2.08 Å and 2.53 Å for the N-terminal domains, 1.53 Å and 2.56 Å for the (α/α)_6_ toroid domains, and 0.85 Å and 2.63 Å for the C-terminal domains of SDT91673.1^GH139^ and AfcA^GH95^, respectively.

### The catalytic −1 subsite of GH139 shares strong similarities to GH95

**Figure 4:**
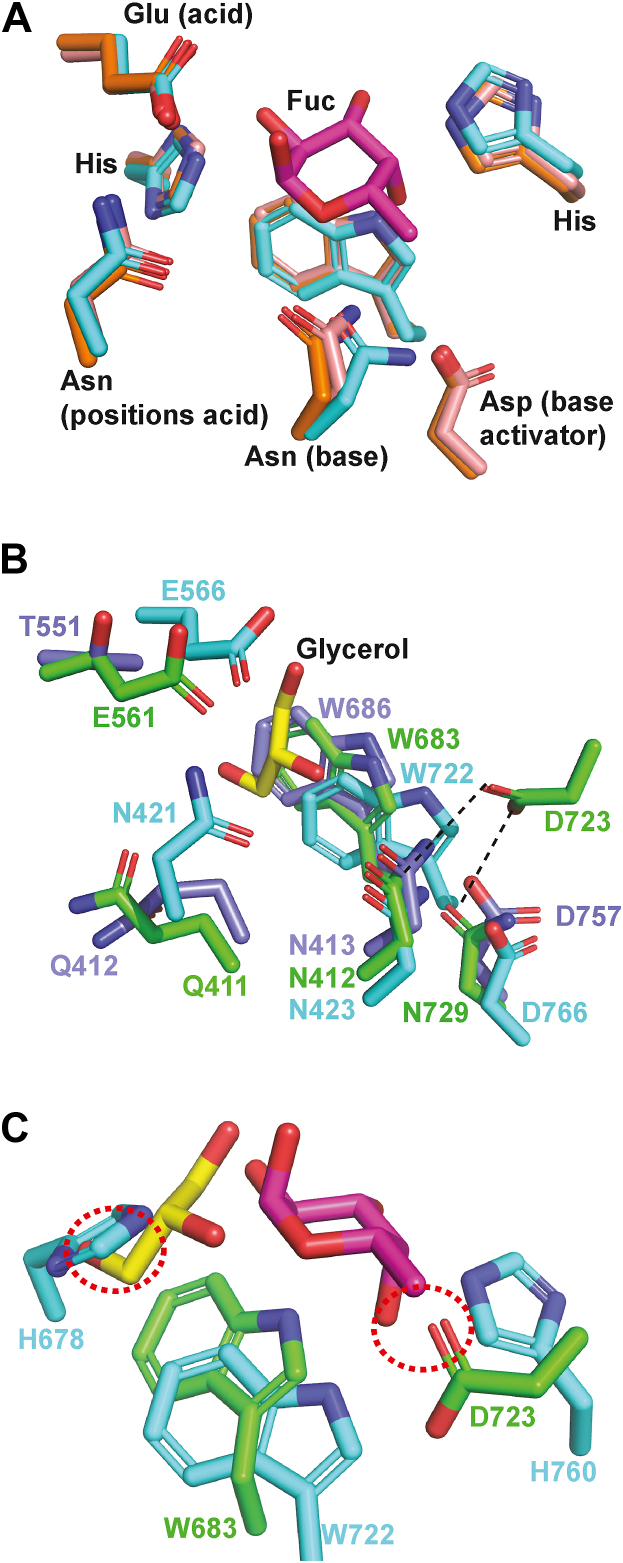
**Architecture of the −1 subsite distinguishes GH139 enzymes from GH95 α-L-fucosidases**. *A*, close-up views of the −1 subsite of three representative GH95s: PDB codes: 2EAE (*cyan*), 4UFC (*pink*), 7KMQ (*orange*). *B*, overlay of the −1 subsite of BT0984^GH139^ (*green*), SDT91673.1^GH139^ (*slate blue*), and AfcA^GH95^ (*cyan*). The glycerol (*yellow*) is from the cryoprotectant of SDT91673.1^GH139^. *C*, overlay of BT0984^GH139^ (*green*) with AfcA^GH95^ (PDB: 2EAE) (cyan) with L-fucose (PDB: 2EAE) (*magenta*) illustrating steric clashes introduced by GH139 residues (*circled, red*) that reshape the pocket and alter substrate specificity.

**Figure 5:**
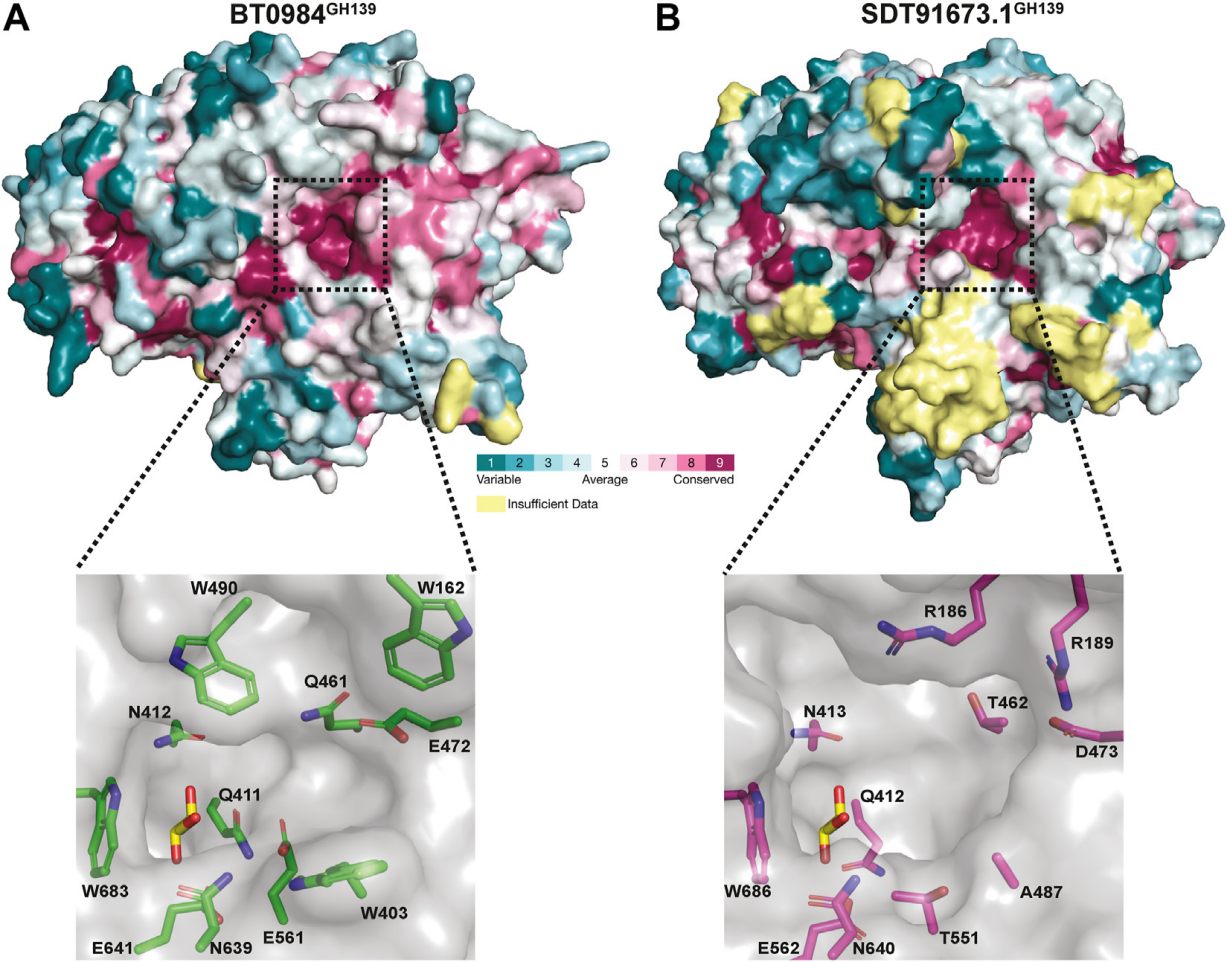
**Identification of the active site of family GH139 enzymes.***A*, consurf analysis of BT0984^GH139^ with a zoom in of the putative active site. *B*, consurf analysis of SDT91673.1^GH139^ with a zoom in of the putative active site.

The GH95 family is known to follow an inverting mechanism with a non-canonical Asn residue proposed as the catalytic base (Fig. 4*A*) (17, 23). Comparison of BT0984^GH139^ and SDT91673.1^GH139^ with the best studied GH95 structural match, AfcA^GH95^ from *B. bifidum* (PDB code 2EAC), shows significant similarities between the −1 subsites of GH95 and GH139 (Fig. 5*B*). Notably, the key −1 subsite Trp residue (W683 in BT0984^GH139^) is conserved in GH95, where it provides aromatic stacking to L-fucose or L-galactose (Fig. 4*B*). However, GH139 diverges significantly in other structural features. GH95 enzymes possess two conserved His residues flanking the Trp, while BT0984^GH139^ lacks these residues; indeed, if they were present, they would clash with the overlaid glycerol molecule (Fig. 4*C*) (used here as a proxy for sugar binding). Instead, D723 occupies a comparable position, which would clash with the *O*4 of L-fucose as bound in GH95. D723 is conserved in >98% of GH139 sequences but is absent in SDT91673.1^GH139^.

GH95 enzymes contain two Asn residues with amide side chains that occupy the same spatial positions as Q411 and N412 of BT0984^GH139^ (Fig. 4, A and B). While Gln, like Asn, has an amide side chain, it is bulkier. In AfcA^GH95^, N423, the residue equivalent to N412 in BT0984^GH139^ is proposed to act as a catalytic base, activated by D766. Meanwhile, N421 in AfcA^GH95^, corresponding to Q411 in BT0984^GH139^, is believed to assist in positioning the catalytic acid E566, equivalent to E561 in BT0984^GH139^. These structural similarities suggest that GH139 enzymes are likely to employ a similar catalytic mechanism to GH95. In BT0984^GH139^, the amide side chain of Q411 is positioned 3.5 Å from the catalytic acid E561, and could fulfill the same role as N421 in AfcA^GH95^ upon substrate binding. Although N412 in BT0984^GH139^ is analogous to N423 in AfcA^GH95^, BT0984^GH139^ lacks an equivalent to D766, the residue that activates N423 in AfcA^GH95^. Instead, BT0984^GH139^ contains N729 in place of D766. Additionally, BT0984^GH139^ has an Asp residue, D723, which is absent in GH95 but conserved across GH139 sequences (Fig. 4, B and C). D723 forms hydrogen bonds with both N412 and N729, at distances of 3.3 Å and 2.9 Å, respectively, suggesting it may fulfill the role of activating N412 to function as a catalytic base. Overall, these features suggest altered, but related, −1 subsite interactions in family GH139 members compared to GH95.

### Comparison of the BT0984^GH139^ and SDT91673.1^GH139^ binding sites

Consurf (24) analysis of both BT0984^GH139^ and SDT91673.1^GH139^ structures demonstrates a pocket of conserved residues, comprising the glycan binding site, located at the center of the (α/α)_6_ toroid, consistent with the comparison with GH95. The pocket in SDT91673.1^GH139^ is notably more open than that of BT0984^GH139^. A glycerol molecule from the cryoprotectant occupies the suspected −1 subsite in SDT91673.1^GH139^, stacking against Trp686 and positioned above N412 and Q411 (Fig. 5). When this glycerol molecule is overlaid onto the structure of BT0984^GH139^, it interacts with the same amino acids, indicating conservation of the −1 subsite. However, significant differences emerge in the putative positive subsites. The SDT91673.1^GH139^ pocket is substantially more open and exhibits less hydrophobicity compared to BT0984^GH139^. The openness of the SDT91673.1^GH139^ pocket is facilitated by residues T462, T551, A487, and D473, which replace Q461, E561, W403, and E472 in BT0984^GH139^. Further distinctions arise from the presence or absence of three aromatic residues. In BT0984^GH139^, a triad of Trp residues, W162, W403, and W490, lines the putative positively numbered subsites, providing aromatic stacking interactions for sugars within the *Δbt0984* oligo. In contrast, these residues are absent in SDT91673.1^GH139^. Specifically, W162 and W490 are spatially replaced by R189 and R186, respectively, introducing a more positively charged character. Additionally, replacement of W403 with Ala opens up the active site and may also reduce hydrophobicity. Together, these structural differences suggest that BT0984^GH139^ and SDT91673.1^GH139^ target different substrates, likely to differ in size and charge.

### GH139 family phylogeny and sequence analysis

**Figure 6:**
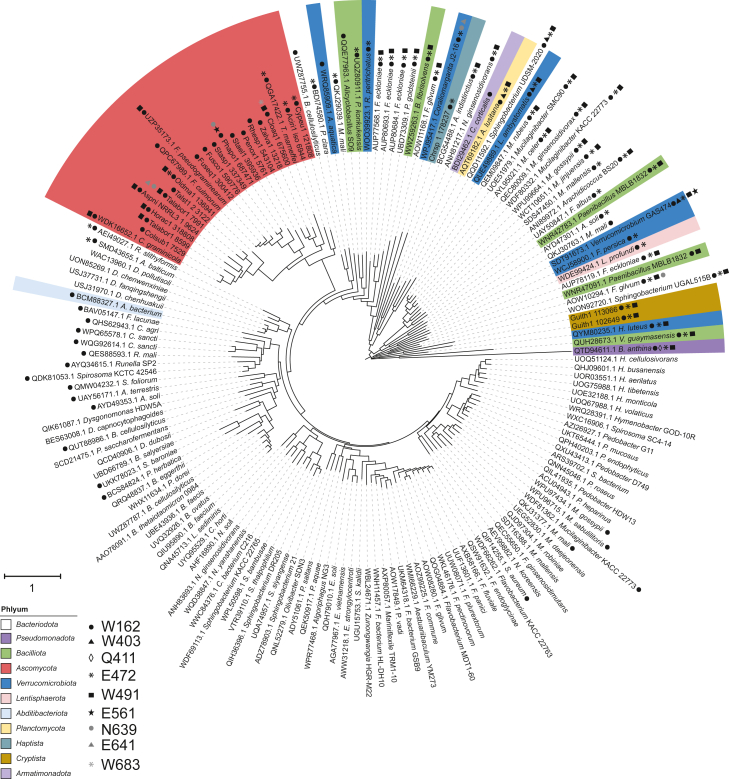
**Phylogeny of GH139 enzymes highlights phylum-level clades and divergence of catalytic residues**. Unrooted maximum-likelihood tree constructed in MEGA X based on alignment of amino acid sequences of each family member obtained from both NCBI and JGI databases. Branch lengths are proportional to the number of substitutions per site; branches are colour coded by phylum. Symbols indicate the lack of conservation of corresponding key residues in homologues of *Bacteroides thetaiotaomicron*. Sequence identifiers have been truncated for clarity.

Phylogenetic analysis revealed that the majority of GH139 sequences analysed, >90%, are from the Bacteroidota phylum. Ascomycota represent the second most abundant phylum (∼7% of sequences), while all other phyla contribute less than 2% each (Fig. 6). The −1 subsite residues, Q411, N412, and W683, are conserved at >99%, with N412, the putative catalytic base, being invariant. E561, the putative catalytic acid, was also highly conserved, appearing in 99% of sequences. The polar/charged residues N639 and E641 are similarly conserved and are present in 98% and 99% of sequences, respectively. The Trp triad residues showed greater variability in conservation, with W162, W490, and W403 present in 45%, 77%, and 98% of sequences, respectively. Interestingly, the complete Trp triad is found in just over 40% of GH139 sequences, indicating some members may rely on alternative substrate recognition.

### Confirmation of key catalytic and substrate binding residues in BT0984^GH139^

**Figure 7:**
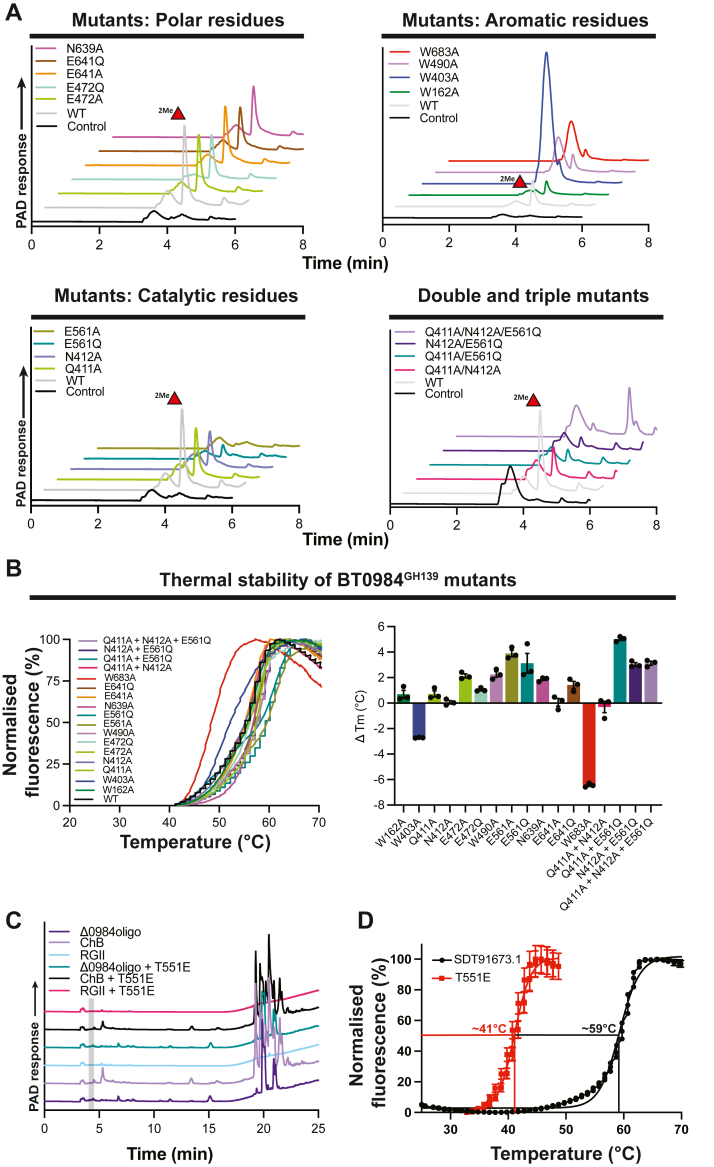
**Catalytic-site mutations alter GH139 activity toward *Δbt0984* oligo and reduce thermal stability**. *A*, HPAEC-PAD traces from overnight endpoint assays comparing wild-type BT0984^GH139^ and its mutant variants (1 μM enzyme, 10 mM MOPS pH 7.0, 150 mM NaCl). *B*, differential scanning fluorimetry of BT0984^GH139^ variants. *Left*: normalized melt curves. *Right*: bar graph of ΔTm mutants *versus* wildtype. *C*, HPAEC-PAD analysis of the SDT91673.1^T551E^ mutant against rhamnogalacturonan ii, isolated chain B, and *Δ0984oligo*; absence of methylfucose release is indicated in the *grey* window. *D*, DSF melt curves of SDT91673.1^GH139^ wild type *versus* the T551E variant, highlighting the destabilizing effect of the substitution.

To confirm the key catalytic and substrate-binding residues in BT0984^GH139^ and SDT91673.1^GH139^, we performed mutagenesis of conserved residues within the predicted active site. This was guided by ConSurf analysis (Fig. 5) and comparison to AfcA^GH95^(Fig. 4). Surprisingly, overnight endpoint assays revealed that only one mutation in BT0984^GH139^, E561A, completely inactivated the enzyme. Mutation of E561 to Gln retained some activity, although this was too low to quantify (Fig. 7*A*; Table 1). Mutations targeting amide-containing side chains in the putative −1 subsite, Q411A and N412A, also resulted in a significant loss of activity on the *Δ0984* oligo. Q411A retained 13.5% of wildtype activity, while N412A could not be quantified (Fig. 7*A*; Table 1). Combining these mutations had a severe effect: the double mutants Q411A/E561Q and N412A/E561Q displayed activity in endpoint assays that was too low to quantify, while the Q411A/N412A double mutant retained 0.03% of wildtype activity (Fig. 7*A*; Table 1).

The Trp triad, which plays a key role in substrate recognition, was also examined through mutagenesis. Mutations in any of the three conserved Trp residues caused major impairments in activity. The W162A mutant was the mildest, showing a 20-fold reduction in activity, whilst W490A and W403A mutants were 1000- and 10,000-fold less active, respectively (Table 1); the relative loss in activity correlates with the conservation level of these residues. The mutation W683A, a residue proposed to provide aromatic stacking interactions with the substrate at the putative −1 subsite, resulted in a 5000-fold loss in activity (Table 1). Such dramatic losses are consistent with the established role of aromatic stacking in glycoside hydrolase function. Unexpectedly, the HPAEC chromatograms for the W403A, W490A, and W683A digestions showed a large contaminating peak immediately before the MeFuc peak that was consistently reproducible but remained unidentified (Fig. 7*A*).

Mutation of E472 to Ala and E641 to Ala or Gln reduced catalytic activity by 100 to 1000-fold (Table 1). The E472Q mutant retained some activity in endpoint assays, but rates could not be quantified (Fig. 7). The mutant N639A resulted in a 100-fold decrease in activity (Table 1). Thermal melt analysis showed that most mutants exhibited increased stability relative to the wild-type enzyme, with Tm values rising by ∼1 °C to 5 °C (Fig. 7*B* and Table S4). Significant exceptions were W403A and W683A, which showed around a ∼3 °C and 6 °C reduction in Tm, respectively, potentially contributing to their loss in activity. The putative catalytic base, N412A, showed no significant difference in Tm relative to wildtype. Ala and Gln mutants of E561, the putative catalytic acid, showed stabilization of ∼3 °C to ∼5 °C compared to wildtype (Fig. 7*B*). This suggests their loss of activity was due to functional impairment rather than structural destabilization. Based on these data, and with comparison to GH95, it appears that N412 and E561 function as the catalytic base and acid, respectively, in an inverting catalytic mechanism.

In SDT91673.1^GH139^, one of the rare (<1%) GH139 sequences that lacks the proposed catalytic acid, E561 (BT0984^GH139^ numbering), this position is instead occupied by T551. Attempts to restore activity by generating a T551E mutant were unsuccessful, with no detectable activity observed against any tested substrates (Fig. 7*C*). Melt curve analysis revealed that the T551E mutant was significantly destabilised, with a 20 °C reduction in Tm relative to the wildtype enzyme (Fig. 7*D*).

### Recognition of the *O*2 methyl group revealed by molecular dynamics

**Figure 8:**
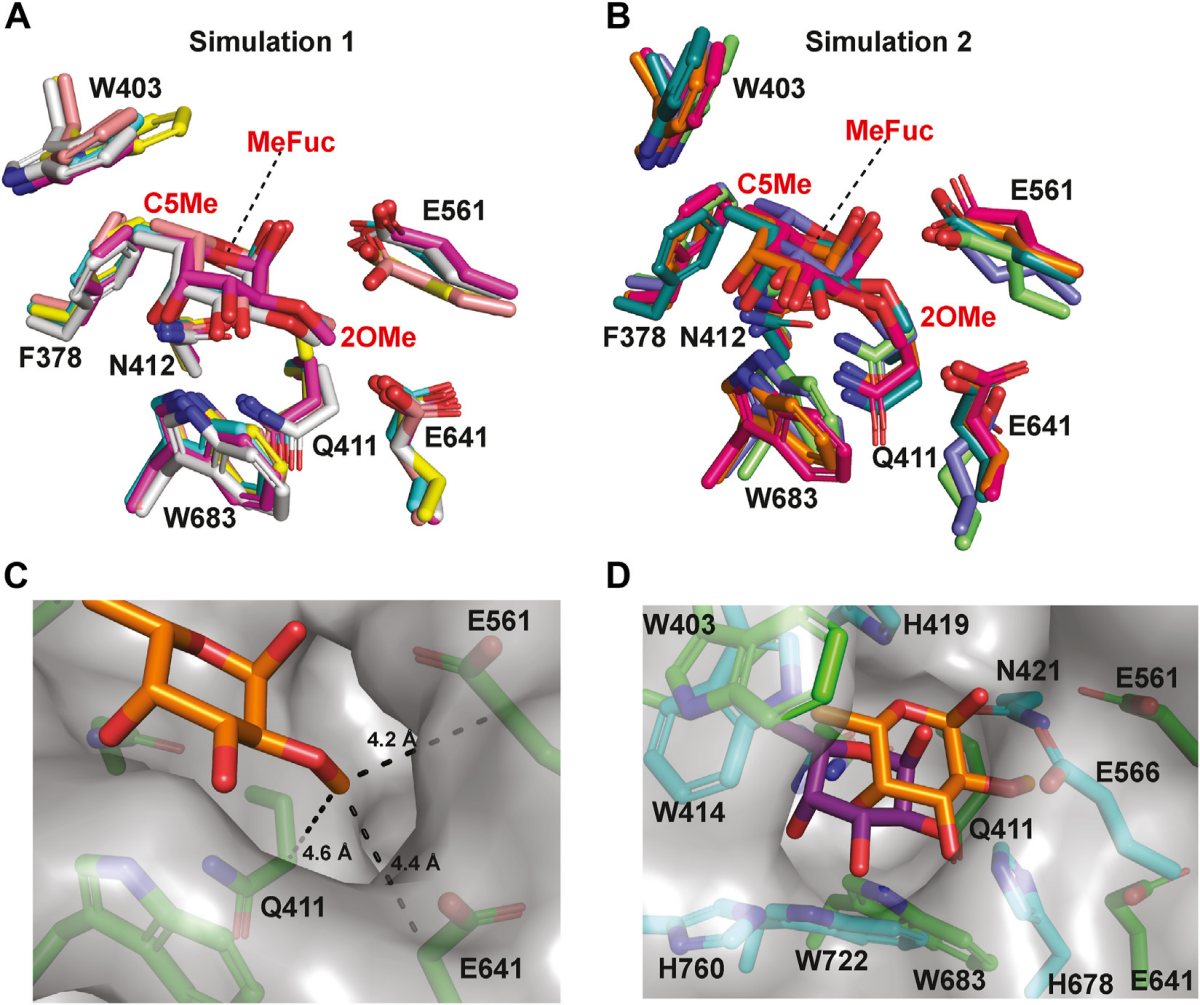
**Molecular dynamics simulations reveal the interaction of *O*2-methyl-α-L-fucose with BT0984^GH139^**. *A*, stick representation of the *top* five binding poses from molecular dynamics simulation one of BT0984^GH139^ in complex with *O*2-methyl-α-L-fucose. *B*, stick representation of the *top* five binding poses from simulation 2. *C*, surface and stick representation of BT0984^GH139^ with *O*2-methyl-α-L-fucose (pose one from simulation one shown) highlighting the proposed *O*2-methyl binding pocket. *D*, overlay of pose 1 (simulation 1), in *green* with an *orange* MeFuc, with family GH95 enzyme AfcA (PDB: 2EAC), in cyan, including α-L-fucose (from PDB: 2EAE), in purple, shown as a surface and stick model to illustrate differences in sugar binding poses.

In the absence of a crystal structure of BT0984^GH139^ complexed with substrate or product, we employed molecular dynamics (MD) simulations to investigate how the *O*2 methyl group of MeFuc is accommodated and contributes to substrate recognition. BT0984^GH139^ was docked with MeFuc, guided by the orientation of the experimentally determined 3D structure of the complex of AfcA^GH95^ with α-L-fucose, and two independent simulations were conducted (Tables S5–S7). From each simulation, the top five binding poses were selected for further analysis (Fig. 8, *A* and *B*). The top five binding poses for each simulation positioned the MeFuc, and its *O*2 methyl, in a similar orientation within the active site, although greater pose variability was noted in simulation 2 (Fig. 8*B*). The MD models suggest that the *O*2 methyl of the MeFuc resides in a shallow pocket formed by the methylene groups of Q411, E561, and E641 (Fig. 8*C*). This interaction is unconventional, while the C5 methyl group of MeFuc engages in more typical hydrophobic interactions with F378 and W403. In AfcA^GH95^, the residue N421 is proposed to help position the catalytic acid; however, in BT0984^GH139^, this role is potentially fulfilled by Q411. Q411 is bulkier and adopts a conformation that arches away from the −1 sugar, exposing its hydrophobic methylene group and creating space for the *O*2 methyl group of MeFuc to slot between Q411 and E561 (the putative catalytic acid). This interaction may assist in positioning the catalytic acid, thereby functionally replacing N421 from AfcA^GH95^, and may also modulate its p*K*_a_ value, providing a rationale for the requirement of MeFuc, rather than L-fucose, at the −1 subsite for catalytic activity. An overlay of pose one from simulation 1 with AfcA^GH95^ (PDB: 2EAC; L-fucose from PDB: 2EAE) reveals preservation of the orientation of the fucose ring, along with a small translation. This orientation enables in-line nucleophilic attack by a water molecule activated by a similar position Asn (412 in BT0984^GH139^/423 in AfcA^GH95^). Notably, the side chains of N421 and H678 in AfcA^GH95^ would sterically hinder the altered binding of MeFuc observed in GH139 and prevent accommodation of the *O*2 methyl group (Fig. 8*D*).

### A proposed model for family GH139 catalysis

**Figure 9:**
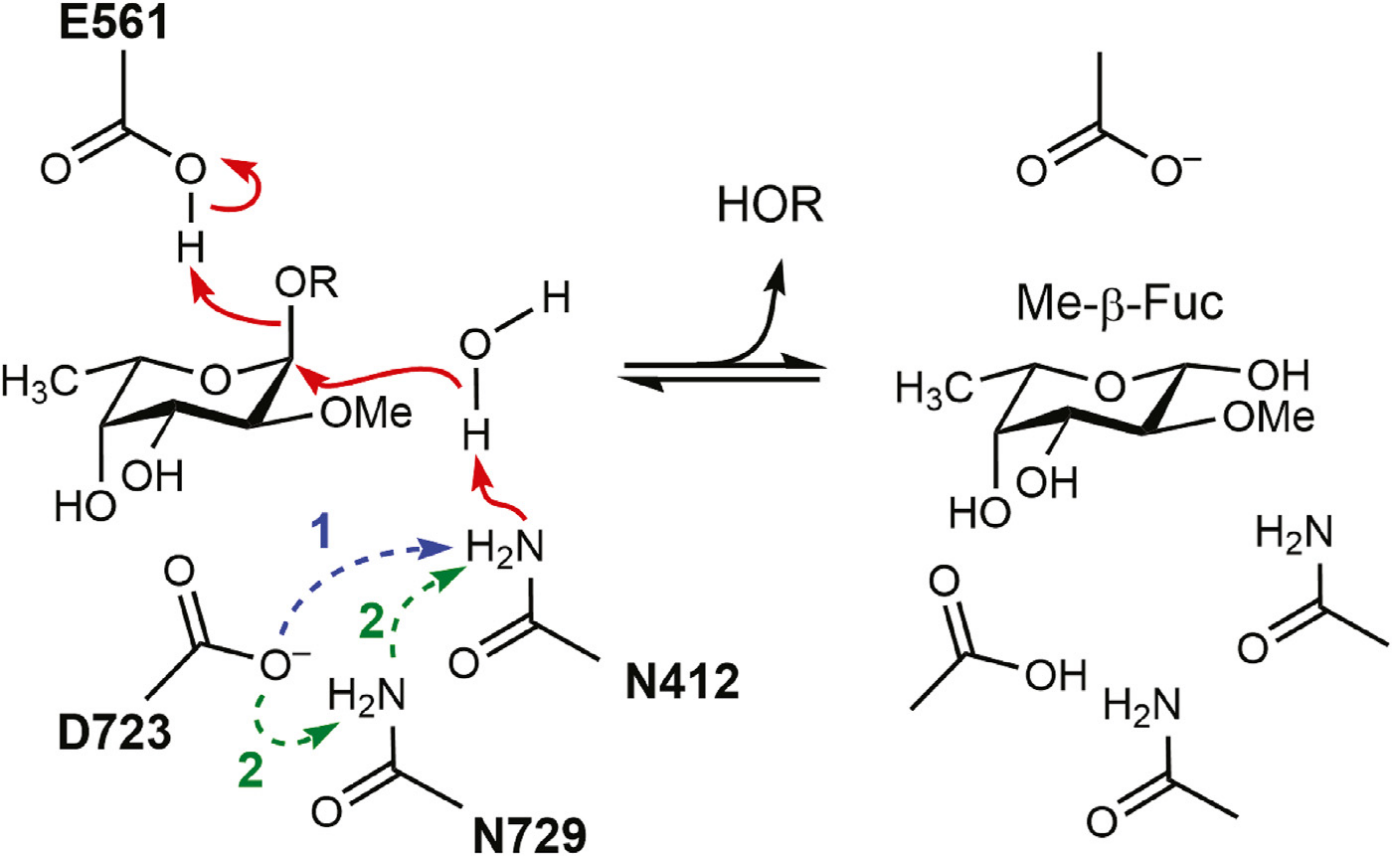
**Two proton-transfer pathways that activate Asn412 for nucleophilic water attack in the GH139 inverting mechanism.** Schematic of the proposed catalytic cycle for BT0984^GH139^, illustrating two alternative routes by which Asn412 is converted into an effective catalytic base (1). Direct pathway. Asp723 abstracts a proton from the side-chain amide of Asn412. The deprotonated Asn412 then removes a proton from the catalytic water, facilitating nucleophilic attack on the anomeric C1 of α-L-fucose (2). Relay pathway. Asp723 deprotonates Asn729; Asn729 subsequently deprotonates Asn412, which in turn activates the catalytic water. This three-step proton relay mirrors the mechanism proposed for inverting GH45 cellulases (26). In both cases reaction results in inversion of configuration and Glu561 acts as the catalytic acid that protonates the leaving group.

Based on our findings, we propose a catalytic model for GH139 enzymes that is analogous to the GH95 family but incorporates distinct structural and mechanistic adaptations. GH139 enzymes operate *via* an inverting mechanism in which we propose that N412 functions as the catalytic base and E561 acts as the catalytic acid (BT0984^GH139^ numbering) (Fig. 9). In this model, N412 deprotonates a water molecule, promoting nucleophilic attack on the anomeric carbon (C1) of MeFuc from the β-face (bottom face). Concurrently, E561 provides general acid assistance by donating a proton to the glycosidic oxygen, facilitating cleavage of the glycosidic bond. For N412 to act as a base, we propose that it must be activated in its imidic acid form, and suggest two possible means (1): direct activation by D723, which acts as a general base; or (2) a proton relay mechanism, in which D723 deprotonates N729, which in turn activates N412. The *O*2 methyl group of MeFuc may play a role in this mechanism by stabilizing the positioning of E561 and/or modulating its p*K*_a_ value, thereby enhancing its capacity to donate a proton during catalysis.

## Discussion

GH139 is the only glycoside hydrolase family known to specifically target α1,2-linked MeFuc. Notably, the conserved Trp triad, important for binding the Δ*0984* oligo, is present in >40% of the GH139 family sequences. This conservation suggests that nearly half of the GH139 family members are adapted to target MeFuc in chain B of RG-II. While no other activities have yet been reported for family GH139, the crystal structure of SDT91673.1^GH139^ reveals a much more open and positively charged pocket that lacks the Trp triad. Interestingly, SDT91673.1^GH139^ belongs to the ∼1% of GH139 sequences that lack a catalytic acid residue, suggesting it may not serve an enzymatic role in *Verrucomicrobium* sp. GAS474 from which it was isolated, but instead may act as a glycan-binding protein for a substrate distinct from chain B of RG-II.

By time-dependent monitoring of the anomeric configuration of MeFuc formed by hydrolysis of the Δ*0984* oligo by BT0984^GH139^, we demonstrated that this enzyme uses a stereochemically inverting catalytic mechanism. Structural and mutagenesis data suggest that BT0984^GH139^ employs a canonical acidic residue as a catalytic acid but a non-canonical Asn as the catalytic base. For this Asn to function effectively, it would need to isomerise to a more basic imidic acid. While unconventional, this suggestion is not unprecedented; families including GH43_24 (25), GH45 (26), and GH95 (17) have also been proposed to use amide-containing side chains as the catalytic base. Among these, family GH45 is the best studied, where an imidic form of Asn acts as a general base. A proton relay, termed a “Newton's cradle”, transfers a proton to the catalytic acid (Asp), enabling proton donation to the glycosidic oxygen (26). A similar mechanism may operate in other GH families, including GH95 and GH139, although further investigation is required to confirm this. We note that while the identity of the catalytic acid in an inverting glycosidase is usually straightforward to establish, it is often more challenging to identify the catalytic base, as in other examples such as GH8c (27, 28) and GH124 (29). Alternate mechanisms for amide activation also exist, requiring a hydrogen bond brace to rotate the amide's nitrogen (30, 31, 32). This rotation supports sp3 hybridization and bulging of the nitrogen's lone pair, thereby increasing nucleophilicity, but no evidence supporting this mechanism was observed in our data.

The −1 subsite of enzymes from families GH95 and GH139 appears similar, but several functional differences are evident. In GH139, the Trp residue responsible for aromatic stacking at the −1 subsite appears to accommodate a translationally shifted binding mode compared to fucose binding in GH95. This shift likely compensates for the presence of D723 in GH139, and the absence of conserved GH95 His residues, ultimately allowing the accommodation of MeFuc, rather than L-fucose, in the GH139 -1 subsite.

In conclusion, these findings provide valuable insights into the catalytic mechanism of BT0984^GH139^, the foundational member of the GH139 family, revealing its shared features with GH95, including the use of an inverting mechanism that employs a non-canonical Asn as the catalytic base. The family is predominantly composed of sequences from the Bacteroidota phylum, with nearly half of these sequences containing the amino acid residues necessary to target MeFuc in RG-II chain B. Notably, structural variation in the pocket architecture among GH139 members suggests a broader range of glycan substrate preferences than currently recognized, warranting further investigation.

## Experimental procedures

### Cloning, protein expression, and purification

The full-length gene encoding BT0984^GH139^ was amplified by PCR using the appropriate primers and the amplified DNA cloned in pET28a using NheI/XhoI restriction sites generating constructs with N-terminal His_6_ tags. Recombinant genes were expressed in *E. coli* strains BL21 (DE3) or TUNER (Novagen), containing the appropriate recombinant plasmid, and cultured to mid-exponential phase in LB supplemented with 50 μg/ml kanamycin at 37 °C and 180 rpm. Cells were then cooled to 16 °C, and recombinant gene expression was induced by the addition of 0.1 mM isopropyl β-D-1-thiogalactopyranoside (IPTG); cells were cultured for another 16 h at 16 °C and 180 rpm. The cells were then centrifuged at 5000 ×*g* and resuspended in 20 mM HEPES, pH 7.4, with 500 mM NaCl before being sonicated on ice (60 cycles of 1 s pulses with 1 s cooling intervals between the pulses, and 30% amplitude). The lysate was then centrifuged at 45,000*g* for 20 min at 4 °C Recombinant BT0984^GH139^ was then purified by immobilized metal ion affinity chromatography using a cobalt-based matrix (Talon, Clontech) and, after a wash with resuspension buffer, eluted with a step gradient of 10-, 50-, and 100-mM imidazole in resuspension buffer. The fractions were then analyzed by SDS-PAGE gel for protein purity and selected fractions dialyzed into 10 mM HEPES pH 7.0 with 150 mM NaCl. If proteins were being carried forward for structural experiments, they were concentrated in centrifugal concentrators (Pierce Protein Concentrator PES: 88,531) with a molecular mass cutoff of 30 kDa and loaded onto a Superdex 200 16/60 size exclusion column. Fractions from this were then subject SDS-PAGE analysis and fractions judged to >95% pure pooled and concentrated in centrifugal concentrators with a molecular mass cutoff of 30 kDa for further downstream structural analyses. Protein concentrations were determined by measuring absorbance at 280 nm using the molar extinction coefficient calculated by ProtParam on the ExPasy server (web.expasy.org/protparam/).

The full-length gene encoding *SDT91673.1*^*GH139*^ from *Verrucomicrobium* sp. was cloned into a pET29a vector using NdeI/HindIII sites (ATG:biosynthetics GmbH, Mezhausen, Germany). The predicted signal peptide from SDT91673.1 sequence (UniProt code: A0A1H2E9C4, residues 23–831) by using SignaIP-6.0 (33) was removed. *E*. *coli* BL21(DE3) cells were transformed with the pET29a-SDT091673.1 plasmid and grown in LB medium supplemented with 50 μg mL^−1^ kanamycin at 37 °C and 200 rpm. Cell cultures were induced at OD_600_ of 0.6 to 0.8 by adding 1 mM IPTG. After 16 h at 18 °C, cells were harvested by centrifugation at 5000×*g* for 30 min at 4 °C and resuspended in 125 ml of 50 mM Tris-HCl, pH 7.5, 500 mM NaCl containing protease inhibitors (Thermo Scientific, A32955) and 3 μl of Benzonase (Merck, 71,205). Cells were lysed by sonication in ice (30 cycles of 10 s pulses with 60 s cooling intervals between the pulses, and 60% amplitude) at 4 °C. The lysate was centrifuged at 20,000*g* for 40 min at 4 °C. The supernatant was filtered through 0.22 μm filters and then applied into a HisTrap HP column (5 ml, Cytiva) previously equilibrated with buffer A. The elution was performed in a ÄKTA Prime Plus system (GE healthcare) using a linear gradient from 0% to 100% of 50 mM Tris pH 7.5, 500 mM NaCl, 500 mM imidazole, for 30 min at 4 ml min^−1^. The purity of the protein was evaluated by SDS-PAGE (TruePAGE Precast Gels, Merck) and protein bands were visualized by Coomassie brilliant blue. For enzymatic activity experiments, the fractions were further purified by size-exclusion chromatography (SEC) using a Superdex 200 10/300 Gl column and 50 mM Tris pH 7.5, 150 mM NaCl as running buffer. SDT091673.1 was concentrated, flash-frozen, and stored at −80 °C. For X-ray crystallography experiments, selected SDT091673.1 fractions were subjected to SEC using the same column equilibrated in 20 mM Tris-HCl pH 7.5.

### Synthesis of 4-nitrophenyl-2-*O*-methyl-α-L-fucopyranoside

Full details of the synthetic procedures can be found in the supporting information.

### Crystallization and data collection

BT0984^GH139^ was concentrated in 10 mM HEPES pH 7.5, 150 mM NaCl using a centrifugal concentrator with a molecular mass cutoff of 30 kDa. Sparse matrix screens were set up in 96-well sitting drop TTP Labtech plates (400-nL drops) using an SPT mosquito crystallization robot. BT0984^GH139^, at a concentration of 5 to 7 mg/ml, gave crystals in a condition containing 20% PEG 3,000, 0.1 M Tris at pH 8.5. These crystals formed in a few days and had a cubic morphology but failed to diffract. Crystals were reproduced and subjected to dehydration by supplementing the reservoir with LiCl_2_ at concentrations ranging from 1 M to 8 M. LiCl_2_ dehydration using concentrations 2 to 4 M gave crystals that diffracted to a resolution of ∼2.8 Å in the orthorhombic space group P2_1_2_1_2_1_. Data were collected at Diamond Light Source (Oxford) on beamline I04-1 (0.98 Å) at 100 K. The data were integrated with XDS (34) and scaled and merged with Aimless (35, 36).

SDT91673.1 was crystallized by mixing 0.25 μl of a protein solution at 6 mg mL^−1^ in 20 mM Tris-HCl pH 7.5 with 0.25 μl of PACT PremierTM screening condition H11, Molecular Dimensions (0.2 M Sodium citrate tribasic dihydrate, 0.1 M Bis-Tris propane pH 8.5 20% and w/v PEG 3350). Crystals grew in 3 to 5 days. All crystals were transferred to a cryo-protectant solution containing 30% glycerol and frozen under liquid nitrogen. Complete X-ray diffraction datasets were collected at beamline i24 (Diamond Light Source (DLS)). SDT91673.1 crystallized in the hexagonal space group P6_5_22 and diffracted to a maximum resolution of 2.05 Å with one molecule in the asymmetric unit (MAU). Datasets were integrated and scaled with XDS following standard procedures (34).

### Structure determination and refinement

The phase problem of BT0984^GH139^ was solved by molecular replacement using the program Phaser (37) or Molrep (38) with a model generated through the RoseTTAfold server (39). Models then underwent recursive cycles of model building in Coot (40) and refinement cycles in Refmac5 (41). The models were validated using Coot (40) and MolProbity (42). Structural Figures were made using Pymol (The PyMOL Molecular graphics system, Version 2.0 Schrodinger, LLC.) and all other programs used were from the CCP4 suite (43). The data processing and refinement statistics are reported in Table S4.

Structure determination of SDT91673.1 crystal form was carried out by molecular replacement methods implemented in Phaser (37) and using the Alphafold two model as a template (44). The final manual building was performed with Coot (40) and refinement with PHENIX refine (45). The structure was validated by MolProbity (42). Data collection and refinement statistics are presented in Table S4. Molecular graphics and structural analyses were performed with the UCSF Chimera package (46).

### Homology modeling and ligand placement

Since no crystal structure of BT0984 in complex with α-2-methoxy fucose was available, a homology model was generated using the glycoside hydrolase family 95 enzyme AfcA from *B. bifidum* (PDB ID: 2EAE) as a structural template, due to its structural, substrate and mechanistic similarities with family GH139 enzymes. The crystal structure of BT0984, as determined in this study, was superposed onto the AfcA template using secondary structure matching (SSM) in COOT(40). Given the low sequence identity between BT0984 and AfcA, further manual adjustments were performed to optimize the alignment of the respective active sites. The α-fucopyranose ligand coordinates from 2EAE were transferred into the BT0984 structure, placing the sugar within the proposed active site of BT0984. This model served as the basis for subsequent docking and molecular dynamics simulations.

### Molecular docking

Docking simulations were performed using the Cresset Flare software suite (47, 48, 49, 50). Protein preparation included addition of polar hydrogen atoms, capping of truncated peptide chains, removal of non-essential water molecules from outside the active site, and energy minimization using the inbuilt structure refinement protocol. The α-fucopyranose, modeled in the active site of BT0984^GH139^, defined the ligand-binding pocket. The docking ligand, 2-methoxy-α-fucose, was prepared by importing its 3D structure and optimizing it using the OpenMM force field (OpenFF and GAFF). Partial atomic charges were computed using the AM1-BCC method. Docking was conducted using Flare's standard precision mode, and the lowest-energy ligand pose was selected for molecular dynamics (MD) simulations.

### Molecular dynamics simulations

MD simulations were carried out using the Flare software platform (47, 48, 49, 50). The system comprised BT0984^GH139^ complexed with the lowest-energy pose of 2-methoxy-α-fucose. Calculations employed the OpenFF force field with explicit TIP3P water molecules. The solvated system was enclosed in a truncated octahedron simulation box with a 10.0 Å solvent buffer. After 200 ps equilibration, a production run of 10 ns was performed using a 4.0 fs timestep, generating 5000 frames. Hydrogen bonding interactions between the ligand and both protein residues and solvent molecules were monitored across the trajectory. A k-means clustering analysis was performed to identify the ten most representative ligand–protein interaction conformers. Of these, the top five clusters (by population) were selected for detailed interaction analysis. The full MD protocol was repeated in an independent run. Ligand binding free energies were calculated for both simulations using the Molecular Mechanics Generalized Born Surface Area (MM-GBSA) method with the GBn2 implicit solvent model. To allow for system stabilization, the first 20% of each trajectory was excluded from free energy calculations.

### Hydrogen bond analysis

Hydrogen bonds between 2-methoxy-α-fucose and BT0984^GH139^ were identified using geometric criteria (distance ≤ 3.5 Å, donor–hydrogen–acceptor angle ≥ 135°). Each hydrogen bond interaction was quantified as a percentage of simulation time across both MD trajectories. For each simulation, the interacting ligand atom, protein residue, and occupancy percentage were tabulated. Both direct protein contacts and water-mediated hydrogen bonds were included in the analysis.

### Site-directed mutagenesis

Site-directed mutagenesis was conducted using a modified PCR-based QuickChange protocol. Appropriate primer pairs with the mutation were designed to have overlapping 5′ regions of 18 bps and 12 bps non-overlapping at the 3′ end (Table S8). These primers, 0.3 μM were mixed with 10 ng of target plasmid and subject to 18 cycles of 95 °C for 30s, 50 °C for 30s, and 72 °C for 4 min; an initial single cycle of 95 °C was done preceding the 18 cycles. CloneAmp HiFi PCR Premix (Takara Bio) was utilised for all PCR reactions. A 0.8% agarose gel was run to visualize successful reactions and to those Dpn1 was added prior to transforming 2 to 5 μl in 100 μl of Top10 super competent cells. These were then plated onto kanamycin containing (50 μg/ml) LB-agar plates and grown overnight at 37 °C. Colonies were then picked, grown up in LB supplemented with 50 μg/ml kanamycin, and plasmid DNA prepped and sequenced to confirm the correct sequence.

### Spectrophotometric linked assays

Kinetics were performed using a Biochrom Libra S22 UV/Vis Spectrophotometer equipped with an eight position water heated cell changer connected to a water circulator set to 37 °C. Reactions were carried out in black walled quartz cuvettes with a 500 μl volume. Reactions were run for 10 min before the addition of the enzyme to ensure a zero baseline was acquired. Enzyme concentrations from 5 nM to 5 μM were deployed. The release of methylfucose by BT0984^GH139^ was monitored *via* a linked assay that couples methylfucose release to the production of NADH from NAD+ which can then be monitored at a wavelength of 340 nm. When BT0984 ^GH139^ releases methylfucose from the Δ0984 oligo, it exposes a D-galactose residue that can be removed by the RG-II specific galactosidase BT0993^GH2^. The released D-galactose is acted on by a galactose mutarotase and dehydrogenase, which removes a proton from D-galactose and adds it to NAD+, to form NADH. The galactose mutarotase and dehydrogenase are purchased as part of a kit from Megazyme (K-ARGA). Specific activity was measured at a substrate concentration of 100 μM. GraphPad Prism 10 software was used to analyze all kinetic data.

### Differential scanning fluorimetry

Thermal shift/stability assays (TSAs) were performed using an Stratagene M x 3005P Real-Time PCR machine (Agilent Technologies) and SYPRO-Orange dye, at a 1:1000 dilution, (emission maximum 570 nm, Invitrogen) with thermal ramping between 20 and 95 °C in 1 °C step intervals per data point to induce denaturation of purified, folded, GH139 enzymes and various mutants. The melting temperature (Tm) corresponding to the midpoint for the protein unfolding transition was calculated by fitting the sigmoidal melt curve to the Boltzmann equation using GraphPad Prism, with R^2^ values of ≥0.99, as described in (51). Data points after the fluorescence intensity maximum were excluded from the fitting. Changes in the unfolding transition temperature compared with the control curve (ΔT_m_) were calculated for each ligand. A positive ΔT_m_ value indicates that the ligand stabilizes the protein from thermal denaturation and confirms binding to the protein. All TSA experiments were conducted using a final protein concentration of 5 μM in 100 mM Bis-Tris-Propane (BTP), pH 7.0, and 150 mM NaCl, supplemented with the appropriate ligand concentration. Three independent assays were performed for each protein and protein ligand combination.

### Thin layer chromatography (TLC)

End point assays were analyzed by TLC by spotting 2 μl of sample onto aluminum-backed silica plates (supleco gel 60 matrix: Z740230–25EA) and resolved in butanol: acetic acid: water (2:1:1) running buffer. The plates were dried, and the sugars were visualized using diphenylamine stain (1 ml of 37.5% HCl, 2 ml of aniline, 10 ml of 85% H_3_PO_3_, 100 ml of ethyl acetate, and 2 g diphenylamine) and heated at 450 °C for 2 to 5 min with a heat gun. Protein buffer conditions were 10 mM MOPS pH 7.0 with 150 mM NaCl, and reactions ran at 37 °C using 1 μM of protein. Polysaccharide substrate concentrations were 5 mg/ml and oligosaccharide concentrations were 5 to 10 mM.

### NMR analysis

All NMR spectra were recorded using a Bruker NEO 700 MHz spectrometer equipped with a 5 mm TCI Prodigy nitrogen cryoprobe. For NMR time-course experiments, a 600 μl sample of the oligosaccharide in D_2_O was prepared. In the case of *Δbt0984* oligo, 0.5 mg was dissolved in 600 μl of D_2_O, while for 4NP-MeFuc, it was at a concentration of 10 mM. Spectra were recorded on this sample before the sample was removed from the magnet and the time course initiated by adding 100 μl of BT0984, which had been buffer exchanged into D_2_O 3 times, at concentrations of 50 μM for the oligo sample and over 80 μM for 4NP-MeFuc, directly to the NMR tube.

Due to the intense HOD signal, presaturation solvent suppression (Bruker pulse sequence “zgpr”) was employed with 64 scans. The first three spectra were acquired consecutively at the start of the time course, followed by a delay to collect subsequent spectra every 10 min. Assignment spectra were recorded both immediately after enzymatic digestion and after removing excess protein *via* SEC. These assignment experiments included a COSY with presaturation solvent suppression, 1D ^13^ C, ^13^C -HSQC, ^13^C -HMBC, and ^13^C -HSQC-TOCSY spectra. The default Bruker parameters were used for all spectra, with an increased number of scans as required, and increases in indirect dimension resolution in several of the 2D spectra. Additionally, a second ^13^C -HMBC spectrum was recorded for the Δbt0984 sample post-filtration, with delays optimized for smaller coupling constants (6 Hz rather than the default 8 Hz). While this adjustment typically reduces sensitivity, it can increase the sensitivity of the experiment to signals relating to smaller ^*n*^J_CH_ couplings.

### High-performance anion exchange chromatography (HPAEC)

To monitor the liberation of MeFuc from the 0984 oligo, the enzyme variants at 5 μM were mixed 1:1 with 2.67 mM of the substrate and incubated overnight at 37 °C. These reaction mixtures were diluted 1:100 in 10 mM Mops 150 mM NaCl, pH 7.0, and analyzed by HPAEC coupled to pulsed amperometric detaction (PAD) for the production of MeFuc. HPAEAC was performed using a Dionex CarboPac PA200 analytical and guard column (Thermo Scientific). The HPEAC elution profile consisted of a 10 min isocratic phase of 100 mM NaOH, followed by a linear gradient to 500 mM NaOAc over 20 min and remaining under these conditions for an additional 10 min. A wash step in 500 mM NaOH was performed for 5 min prior to re-equilibration in 100 mM NaOH for 15 min.

### Phylogenetic and sequence analyses

All members of Glycoside hydrolase family 139 were downloaded from the CAZy database. Sequences were aligned using MAFFT version 7.0 under default settings (52). Following the initial alignment, the dataset was further refined to remove redundant sequences with more than 90% similarity using CD-HIT. The remaining 161 sequences were realigned and imported into MEGA (ver 10.2.6) (53). A maximum likelihood statistical method was used with a WAG + G model with five discrete categories and 100 bootstrap replicates. All other settings were left as default. The tree was visualized and annotated with phyla and differential residue information using iTOL.

## Data availability

The crystal structure datasets generated have been deposited in the Protein Data Bank (PDB) under the following accession numbers: 9HYQ and 9HMB. Information on all other data and materials is contained within the main manuscript and Supplemental Information.

## Supporting information

This article contains supporting information (54, 55, 56, 57).

## Code availability

No new codes were developed or compiled in this study.

## Conflict of interest

The authors declare that they have no conflicts of interest with the contents of this article.
