# Supplementary material for: Understanding the substrate recognition and catalytic mechanism of 2-O-methyl fucosidases from glycoside hydrolase family 139
Source: J Biol Chem. 2025 Jun 20;301(8):110407. doi: 10.1016/j.jbc.2025.110407 (PMC12282258; doi:10.1016/j.jbc.2025.110407)
Supplement: Supporting information [file mmc1.docx]

**SUPPLEMENTAL INFORMATION**

**Structural and functional insights into methyl fucosidases from glycoside hydrolase family 139**

**Zak McIver^1,2,3#^, Alicia Moraleda-Montoya^4,#^, Zongjia Chen^5^, Ruwan Epa^5^ David Starns^6^, Matthew Davy^7^, Mikel García-Alija^4^, Arnaud Basle^8^, Mario Schubert^9^, Didier Ndeh^10^, Beatriz Trastoy^4,11^, Spencer J. Williams^5^, Marcelo E. Guerin^12^, and Alan Cartmell^1,2,3^***

^1^Department of Biology, University of York, York, YO10 5DD, United Kingdom,

^2^York Structural Biology Laboratory, ^3^York Biomedical Research Institute, University of York, York, YO10 5DD, United Kingdom

^3^York Biomedical Research Institute, University of York, York, YO10 5DD, United Kingdom,

^4^Structural Glycoimmunology Laboratory, Biobizkaia Health Research Institute, 48903 Barakaldo, Spain.

^5^School of Chemistry and Bio21, Molecular Science and Biotechnology Institute, University of Melbourne, Parkville, Victoria3010, Australia

^6^School of Molecular and Cellular Biology, Faculty of Biological Sciences, University of Leeds

^7^ Department of Chemistry, University of York, York, YO10 5DD, United Kingdom,

^8^Newcastle University Biosciences Institute, Medical School, Newcastle University, Newcastle upon Tyne NE2 4HH, UK

^9^ FU Berlin, Department of Biology, Chemistry and Pharmacy, Takustr. 3, 14195 Berlin, Germany

^10^ School of Life Sciences, E109, The James Hutton Institute, Errol Road, Invergowrie, Dundee, DD2 5DA, University of Dundee

^11^Ikerbasque, Basque Foundation for Science, 48009 Bilbao, Spain.

^12^Structural Glycobiology Laboratory, Department of Structural and Molecular Biology; Molecular Biology Institute of Barcelona (IBMB), Spanish National Research Council (CSIC), 08028 Barcelona, Catalonia, Spain.

^#^These authors contributed equally

*To whom correspondence should be addressed: alan.cartmell@york.ac.uk

**Synthesis of 4-Nitrophenyl-2-*O*-methyl-α-L-fucopyranoside**

**4-Nitrophenyl 3,4-*O*-isopropylidene-α-L-fucopyranoside (1)**

A mixture of 4-nitrophenyl 3,4-*O*-isopropylidene-α-L-fucoside (320 mg, 1.1 mmol), *p*-TsOH (5 mg, 0.03 mmol) and 2,2-dimethoxypropane (420 mg, 4.0 mmol) in DMF (5 mL) was stirred under a N_2_ atmosphere at 80 °C for 1 h. The solvent was removed and the residue was purified by flash chromatography (n-hexane:EtOAc=4:1) to give the product as an oil (332 mg, 91%).

[α]_D_ = -199.82 (*c* = 1.00, CHCl_3_)

¹H ^1^H NMR (500 MHz, CDCl_3_) δ 1.30 (d, *J* = 6.6 Hz, 3H), 1.38 (s, 3H), 1.55 (s, 3H), 4.02 (dd, *J* = 6.6, 3.6 Hz, 1H), 4.14 (dd, *J* = 6.0, 2.3 Hz, 1H), 4.20 (qd, *J* = 6.6, 2.2 Hz, 1H), 4.40 (t, *J* = 6.3 Hz, 1H), 5.61 (d, *J* = 3.6 Hz, 1H), 7.18 (d, *J* = 9.3 Hz, 2H), 8.20 (d, *J* = 9.3 Hz, 2H).

^13^C NMR (101 MHz, CDCl_3_) δ 16.4, 26.0, 27.9, 65.8, 69.3, 75.4, 75.8, 96.7, 109.8, 116.6, 126.0, 142.8, 161.6.

HRMS (ESI^+^) calcd for C_15_H_19_ClNO_7_ [M+Cl]^-^ 360.0850. Found 360.0854.

**4-Nitrophenyl 3,4-*O*-isopropylidene-2-*O*-methyl-α-L-fucopyranoside (2a)**

A mixture of **1** (150 mg, 0.46 mmol) and Ag_2_O (871 mg, 3.76 mmol) in CH_3_I (1 mL) was stirred at room temperature for 8 h (***). The mixture was filtered through Celite, and the Celite was washed thoroughly with EtOAc. The residue was purified by chromatography (n-hexane:EtOAc = 5:1) to give the product as a white crystalline solid (92 mg, 58%).

[α]_D_ = -162.2 (*c* = 1.00, CHCl_3_)

^1^H NMR (500 MHz, CDCl_3_) δ 1.31 (d, *J* = 6.6 Hz, 3H), 1.38 (s, 3H), 1.58 (s, 3H), 3.55 (dd, *J* = 7.6, 3.4 Hz, 1H), 3.57 (s, 3H), 4.05–4.14 (m, 2H), 4.43 (dd, *J* = 7.5, 5.6 Hz, 1H), 5.66 (d, *J* = 3.4 Hz, 1H), 7.19 (d, *J* = 9.2 Hz, 2H), 8.20 (d, *J* = 9.2 Hz, 2H).

^13^C NMR (151 MHz, CDCl_3_) δ 16.4, 26.4, 28.4, 59.3, 65.0, 75.5, 75.8, 78.6, 95.3, 109.4, 116.5, 125.9, 142.7, 161.8.

HRMS (ESI^+^) calcd for C_16_H_22_NO_7_ [M+H]^+^ 340.1391. Found 340.1385.

*** Caution! Prolonging the reaction beyond 16 hours will result in rearrangement to the methyl 2-O-nitrophenyl-fucoside **2b** (9% yield by HPLC separation).

**Methyl 3,4-*O*-isopropylidene-2-(4-nitrophenyl)-α-L-fucopyranoside (2b)**

[α]_D_ = -122.5 (*c* = 0.50, CHCl_3_)

^1^H NMR (600 MHz, CDCl_3_) δ 1.34 (s, 3H), 1.45 (d, *J* = 6.6 Hz, 3H), 1.53 (s, 3H), 3.46 (s, 3H), 3.93 (qd, *J* = 6.5, 2.2 Hz, 1H), 4.10 (dd, *J* = 5.6, 2.2 Hz, 1H), 4.25–4.21 (m, 1H), 4.30–4.27 (m, 1H), 4.31 (d, *J* = 7.9 Hz, 1H), 7.11 (d, *J* = 9.3 Hz, 2H), 8.14 (d, *J* = 9.3 Hz, 2H).

^13^C NMR (151 MHz, CDCl_3_) δ 16.5, 26.1, 28.1, 57.0, 69.1, 76.5, 78.2, 80.7, 102.4, 110.2, 116.5, 125.7, 141.9, 164.3.

HRMS (ESI^+^) calcd for C_16_H_22_NO_7_ [M+H]^+^ 340.1391. Found 340.1390.

**4-Nitrophenyl 2-*O*-methyl-α-L-fucopyranoside (3)**

A solution of **2a** (25 mg, 0.074 mmol) in CH_2_Cl_2_ (2 mL) was cooled in an ice bath, and TFA (19 μL, 0.26 mmol) was added under a N_2_ atmosphere. The mixture was warmed to room temperature and stirred for 4 h. The reaction was neutralized with NEt_3_, and the solvent was removed under reduced pressure. The residue was purified by chromatography (n-hexane:EtOAc = 1:4) to give the product as a white foam (20 mg, 91%).

[α]_D_ = -98.9 (*c* = 0.88, CHCl_3_)

^1^H NMR (500 MHz, CDCl_3_) δ 1.27 (d, *J* = 6.6 Hz, 3H), 2.49 (s, 1H), 2.71 (d, *J* = 3.0 Hz, 1H), 3.50 (s, 3H), 3.72 (dd, *J* = 9.8, 3.4 Hz, 1H), 3.90 (s, 1H), 3.97 (q, *J* = 6.6 Hz, 1H), 4.16 (dt, *J* = 9.8, 3.0 Hz, 1H), 5.78 (d, *J* = 3.4 Hz, 1H), 7.21 (d, *J* = 9.2 Hz, 2H), 8.22 (d, *J* = 9.2 Hz, 2H).

^13^C NMR (126 MHz, CDCl_3_) δ 16.2, 58.5, 67.4, 69.5, 71.2, 77.6, 94.6, 116.6, 126.0, 142.8, 161.9.

HRMS (ESI^+^) calcd for C_13_H_18_NO_7_ [M+H]^+^ 300.1078. Found 300.1073.

**4-Nitrophenyl 3,4-*O*-isopropylidene-α-L-fucopyranoside (1)**

**^1^H NMR**


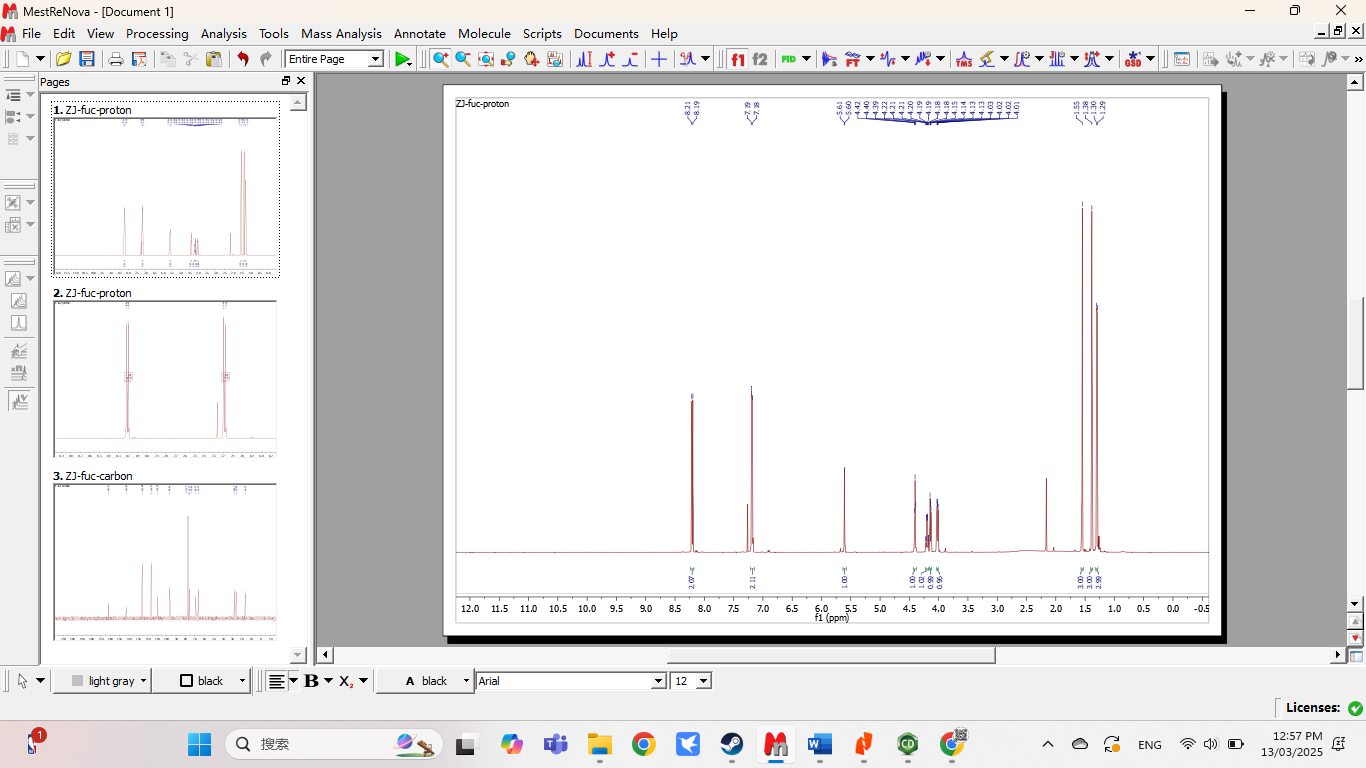


**^13^C NMR**


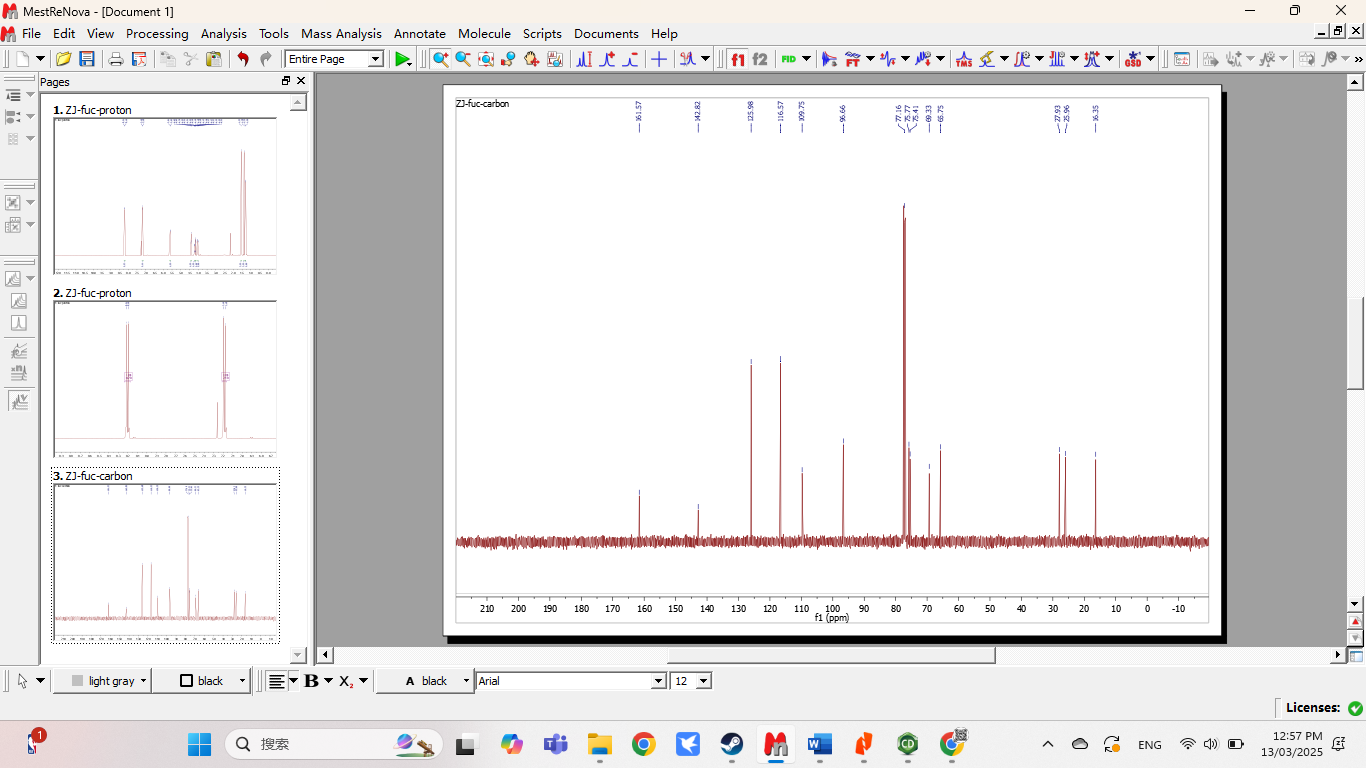


**4-Nitrophenyl 3,4-*O*-isopropylidene-2-*O*-methyl-α-L-fucopyranoside (2a)**

**^1^H NMR**


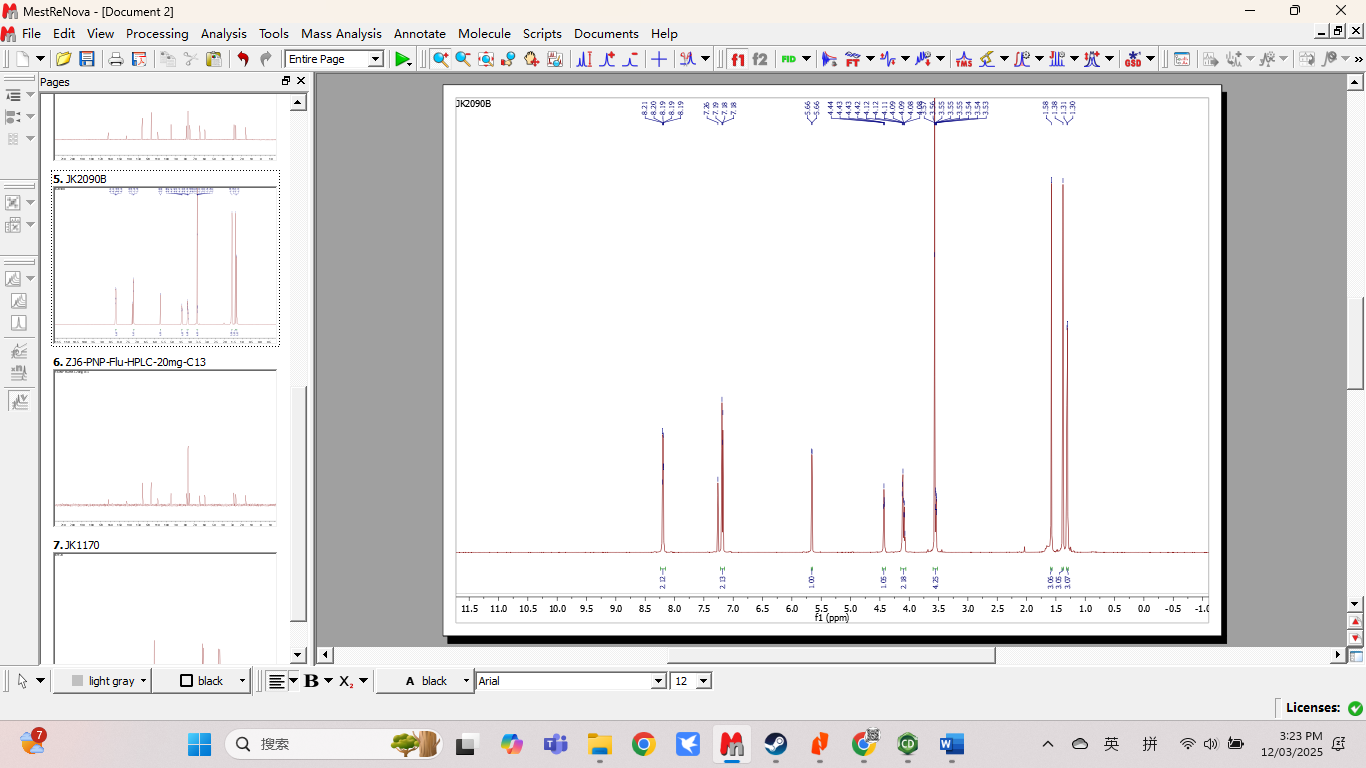


**^13^C NMR**


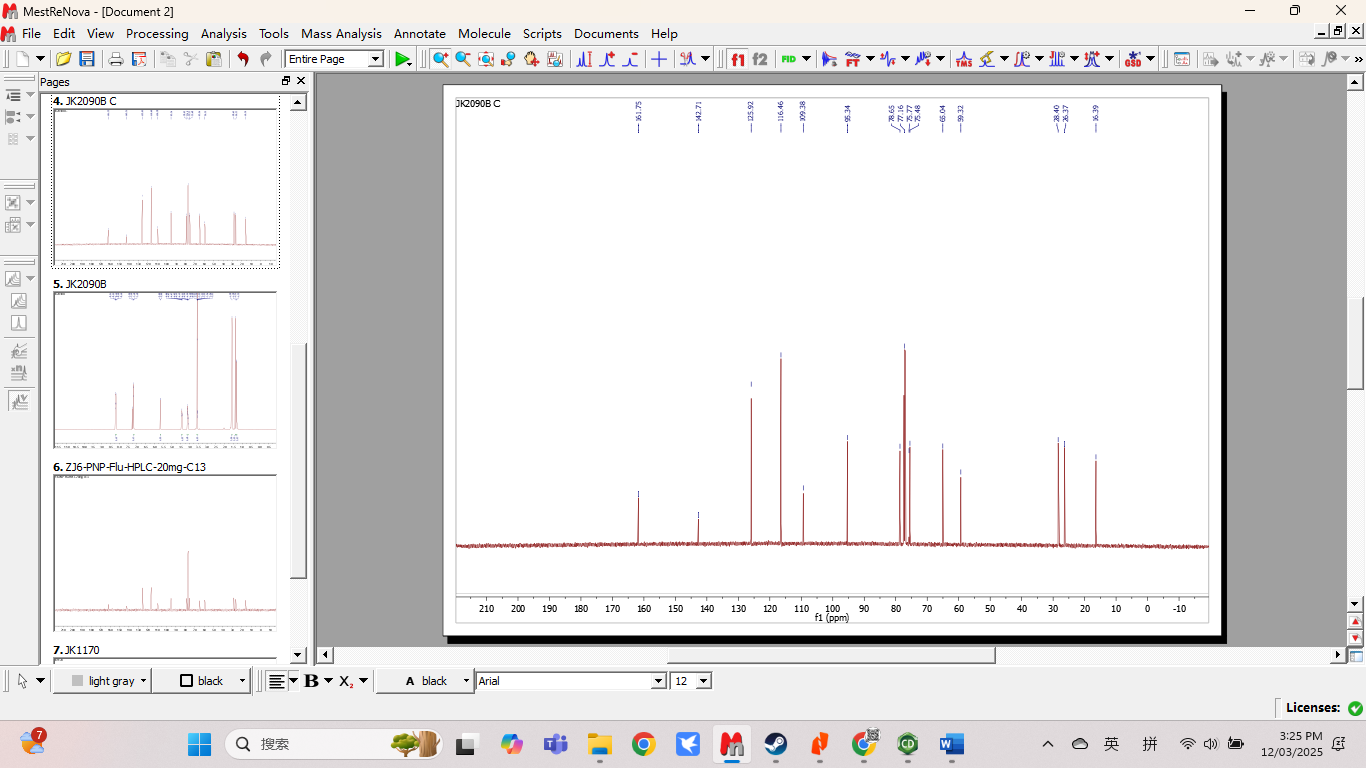


**Methyl 3,4-*O*-isopropylidene-2-(4-nitrophenyl)-α-L-fucopyranoside (2b)**

**^1^H NMR**


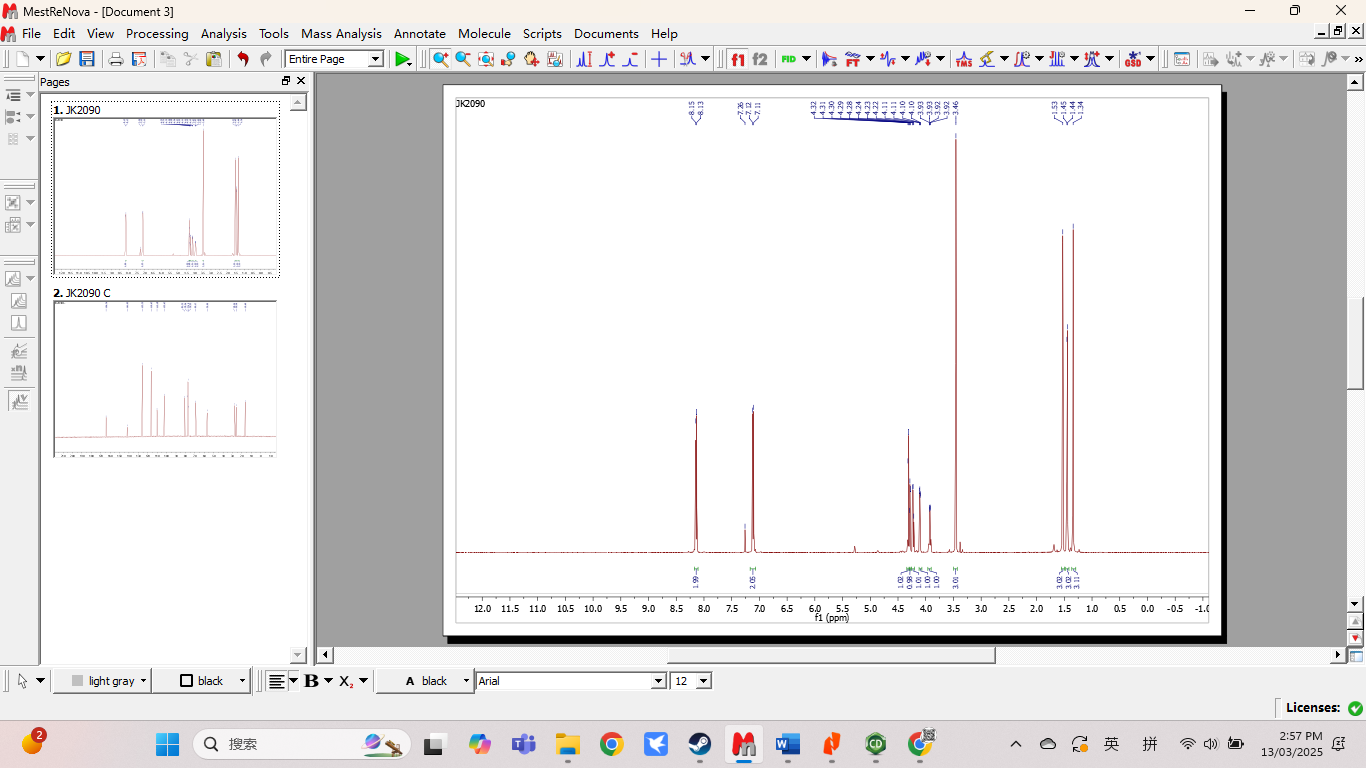


**^13^C NMR**


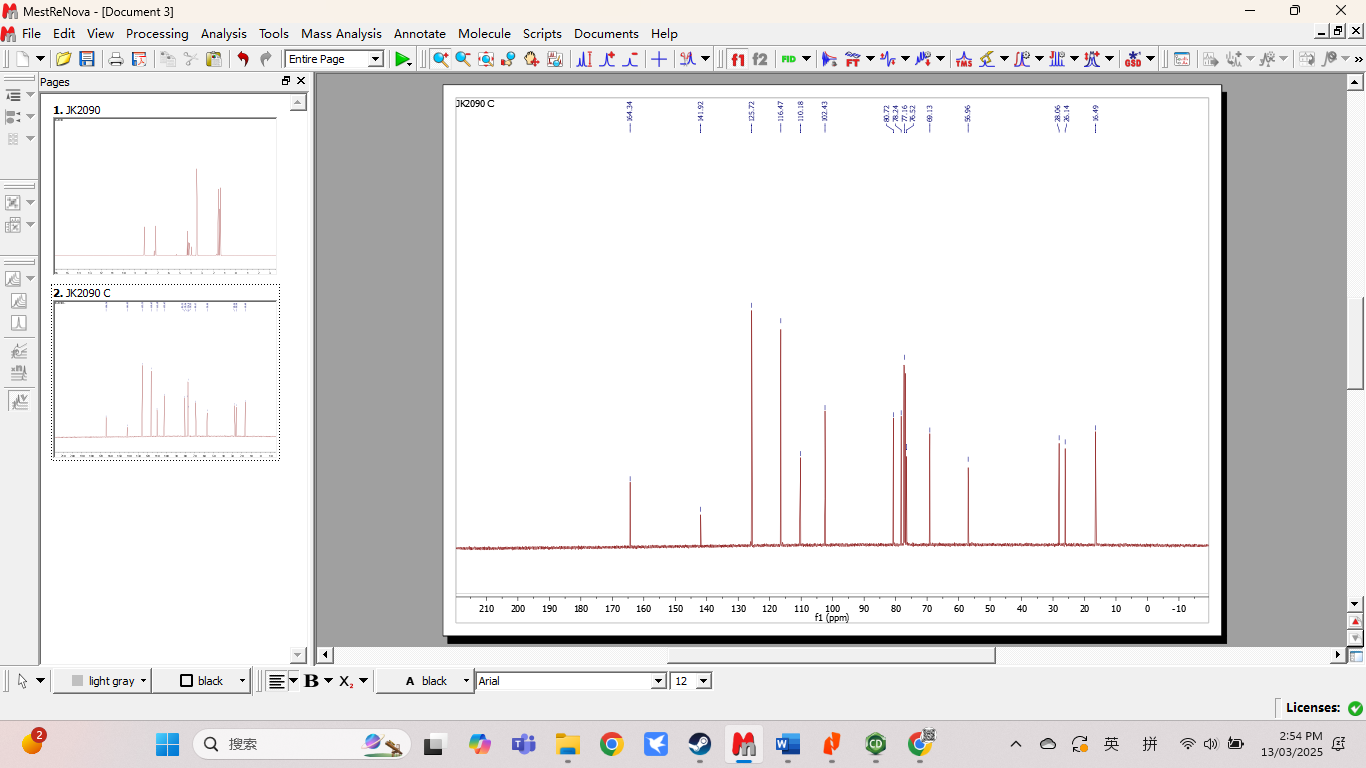


**4-Nitrophenyl 2-*O*-methyl-α-L-fucopyranoside (3)**

**^1^H NMR**


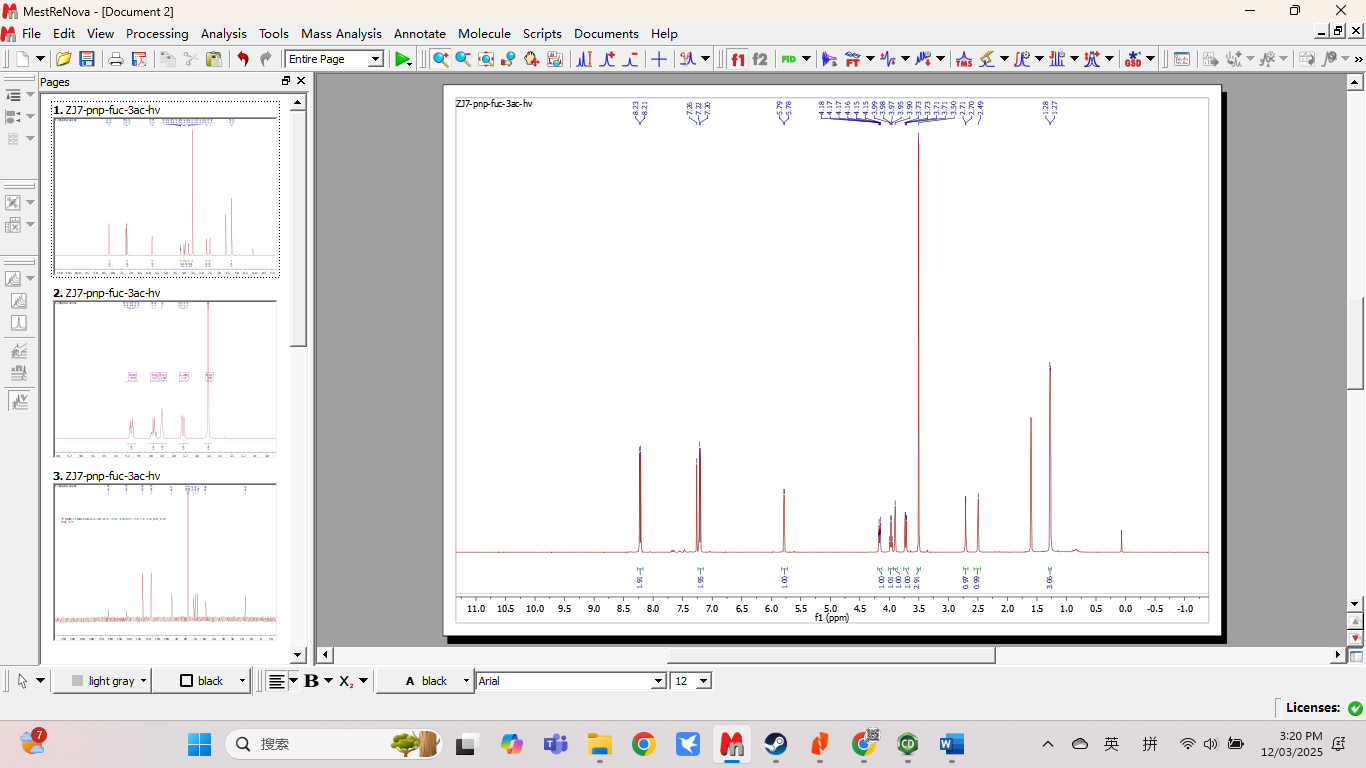


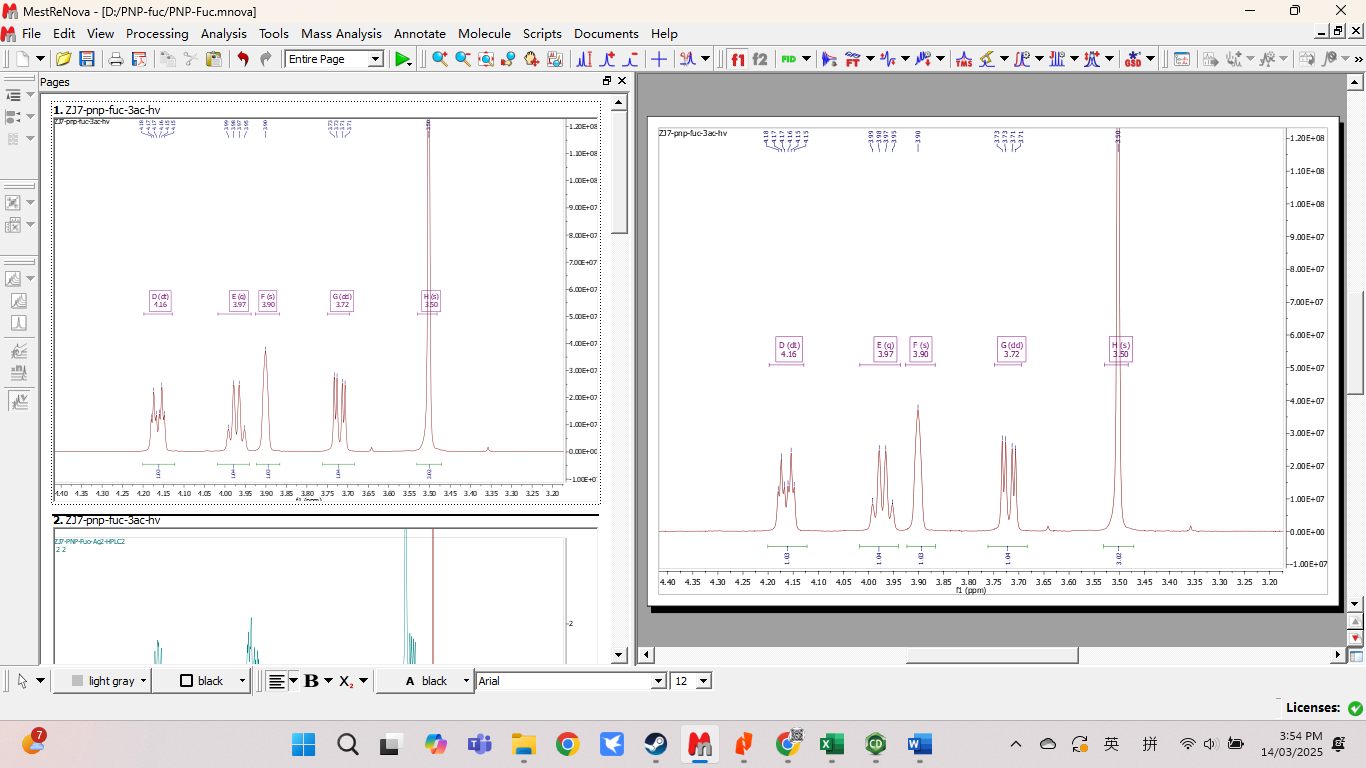


**^13^C NMR**


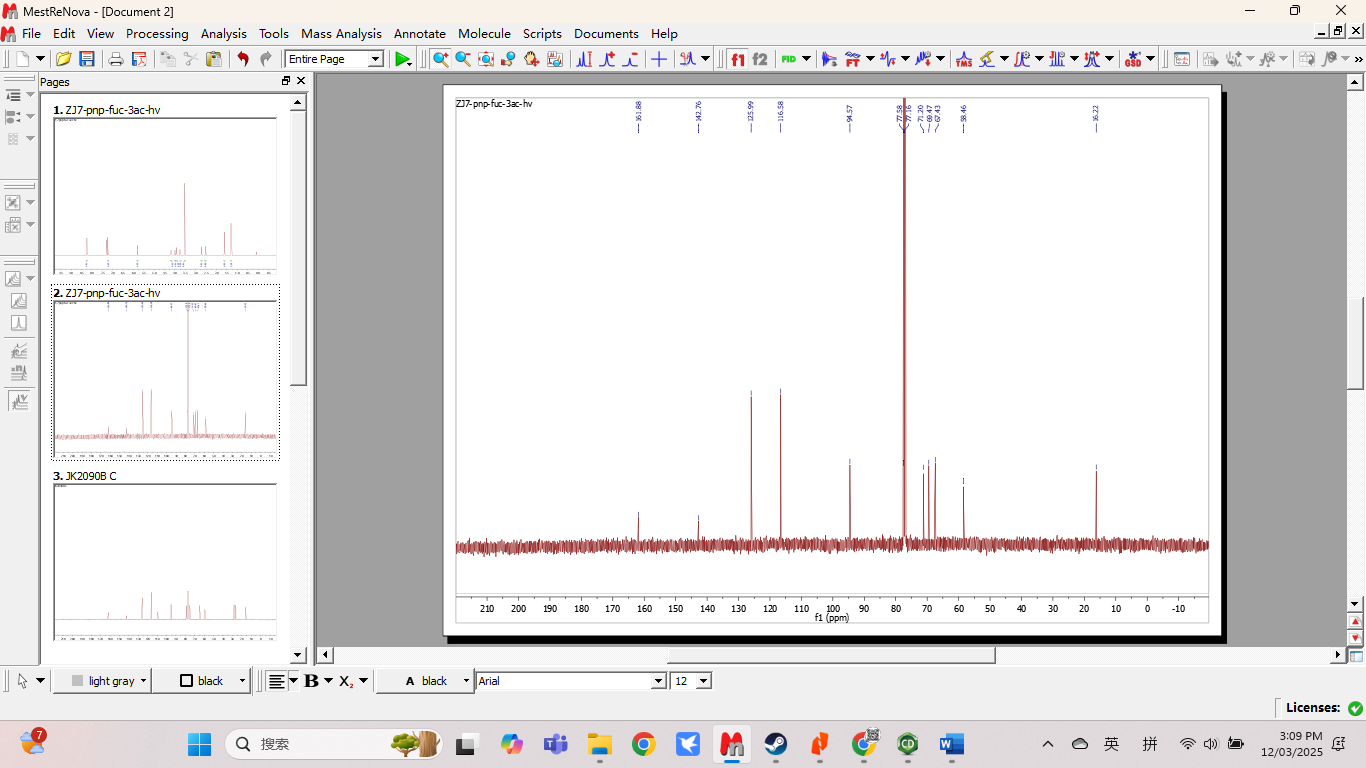


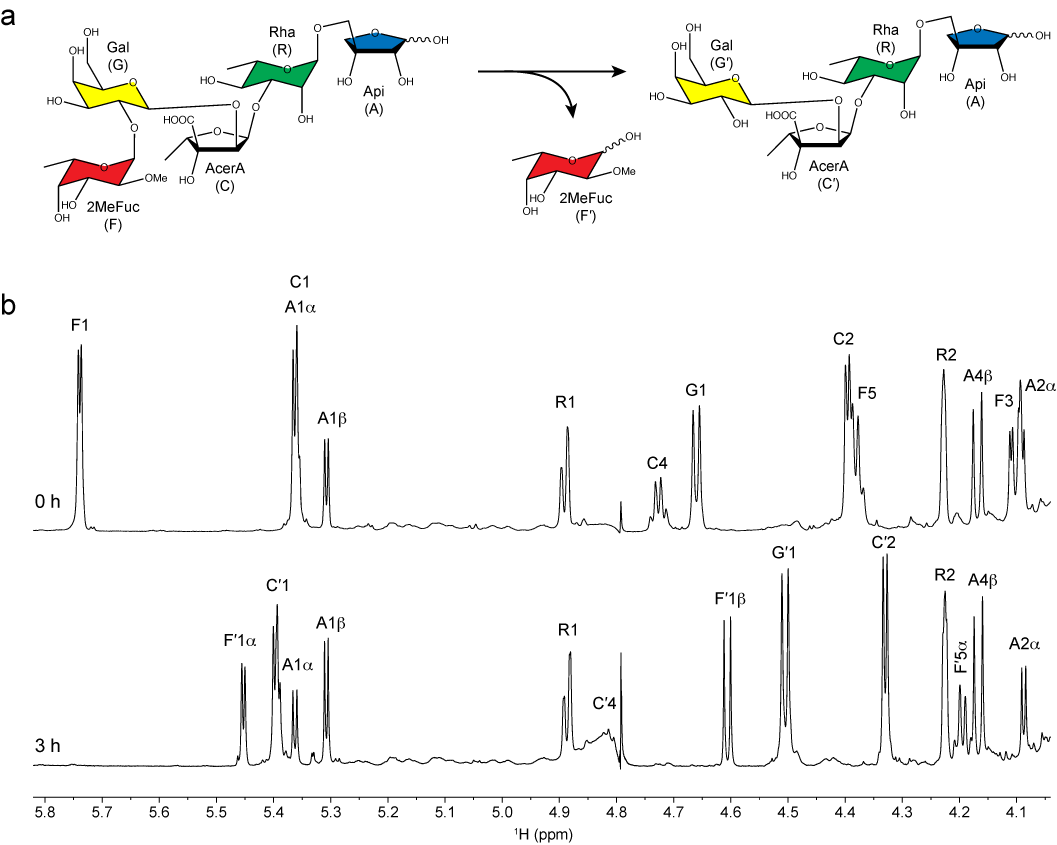


**Supplementary Figure S1**. Chemical shift assignment of the ^1^H 1D spectra of Δ0984 oligo before and after enzymatic treatment. (a) Chemical structure of Δ0984 oligo and the products of the enzymatic cleavage with used nomenclature. (b) ^1^H 1D spectra of Δ0984 oligo before (top) and after enzymatic treatment (bottom) with assignment. Capital letters denote the building block, numbers proton number and in case of a free reducing end α and β denotes the anomer, e.g. F'1α stands for H1 of the free fucose α anomer.


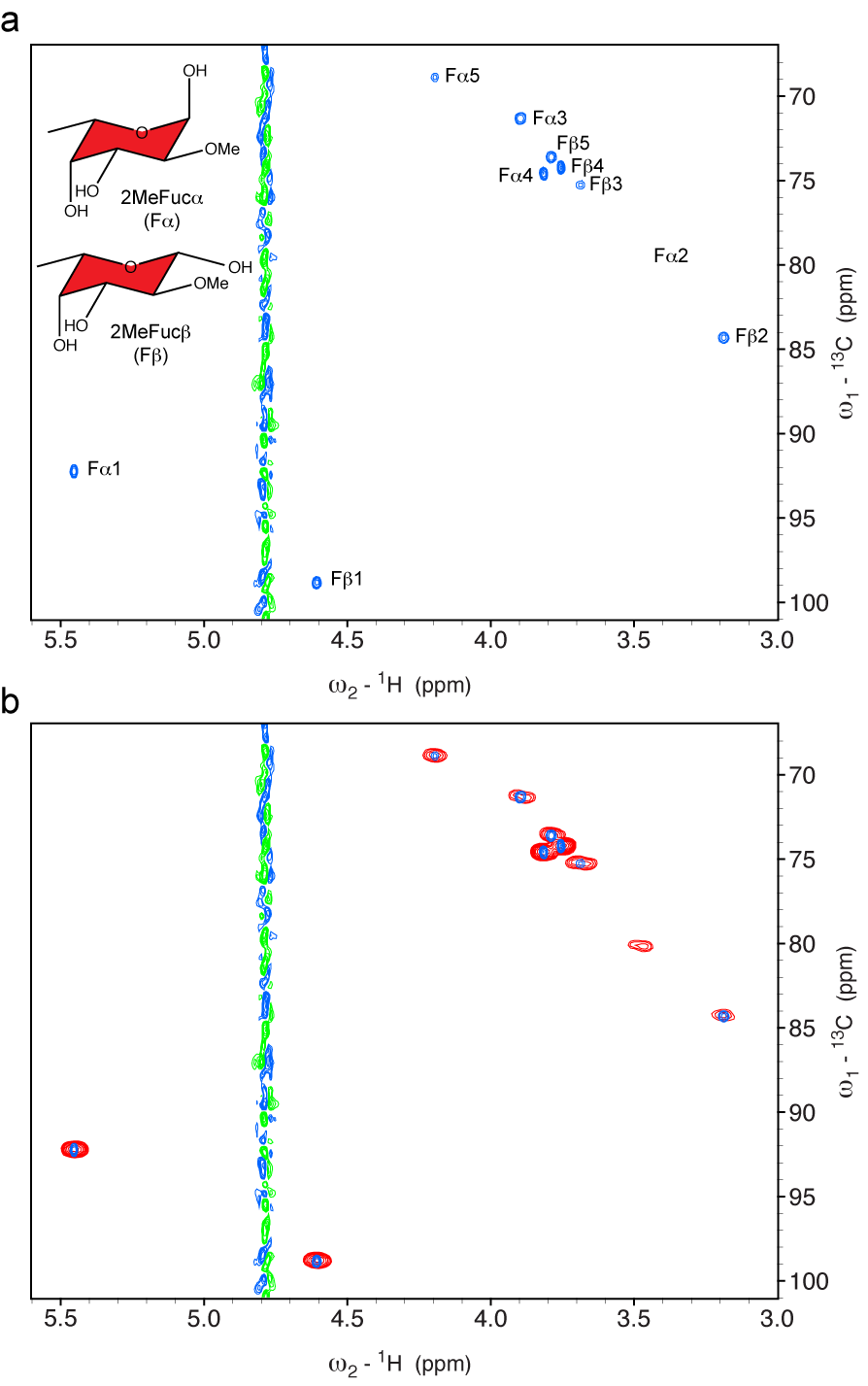


**Supplementary Figure S2**. NMR spectra confirm the released carbohydrate is free 2MeFuc. (a) ^1^H-^13^C HSQC of the released carbohydrate with assignments. (b) Same spectrum as before (blue) overlayed on a ^1^H-^13^C HMQC spectrum of a reference sample of 2MeFuc (red) showing identical peak positions.

**
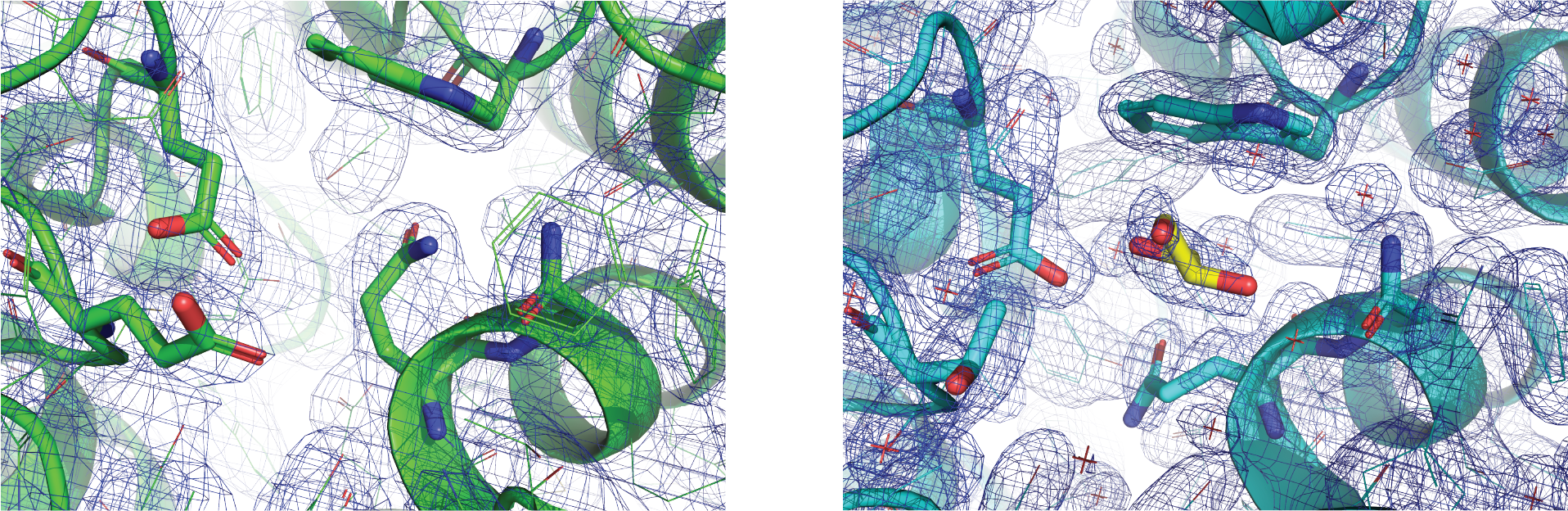
**

**Supplementary Figure S3**. Electron density maps of the GH139 active sites.

Weighted 2Fobs-Fc electron density maps of the active site residues of BT0984, left (2.7 Å), and SDT91673.1. right (2.05 Å). Both maps are contoured at 1σ.

**Table S1** Observed chemical shifts of the Δ0984 oligo measured at 298 K in 10 mM HEPES, with 150 mM NaCl, buffer referenced to DSS in comparison with related structures reported earlier (1-4).

| Moiety | H1 | H2 | H3 | H4 | H4' | H5 | H6/H5' | H7 | C1 | C2 | C3 | C4 | C5 | C6 | C7 |  |
| --- | --- | --- | --- | --- | --- | --- | --- | --- | --- | --- | --- | --- | --- | --- | --- | --- |
| β-Api ^a^ | 5.294 | 3.954 | ⎯ | 4.155 | 3,876 | 3.828 | 3.558 | ⎯ | 104.4 | 80.1 | 80.8 | 75.7 | 71.9 | ⎯ | ⎯ | this work |
|  | 5.233 | 4.158 | ⎯ | 4.348 | 3.935 | 3.986 | 3.655 | ⎯ | 112.5 | n.d. | n.d. | 77.3 | 73.1 | ⎯ | ⎯ | Vidal ^b^ |
|  | 5.12 | n.d. | ⎯ | 4.23 | 3.81 | 4.08 | 3.53 | ⎯ | 112.5 | n.d. | n.d. | 76.9 | 73.3 | ⎯ | ⎯ | Rodriguez^c^ |
|  | 5.09 | 4.12 | ⎯ | 4.16 | 4.01 | 3.72 | 3.72 | ⎯ | 110.9 | 78.0 | 80.8 | 75.0 | 65.0 | ⎯ | ⎯ | Ishi ^d^ |
| α-Api ^a^ | 5.342 | 4.089 | ⎯ | 3.792 | 3.527 | 3.986 | 3.989 | ⎯ | 99.0 | 76.0 | 79.2 | 75.6 | 72.5 | ⎯ | ⎯ | this work |
|  | 5.11 | 4.12 | ⎯ | 4.16 | 4.12 | 3.72 | 3.72 | ⎯ | 104.7 | 73.2 | 78.4 | 75.1 | 65.9 | ⎯ | ⎯ | Ishi ^d^ |
| Rha | 4.878 | 4.222 | 3.938 | 3.626 | ⎯ | 3.783 | 1.325 | ⎯ | 102.5 | 69.3 | 78.1 | 72.9 | 71.3 | 19.5 | ⎯ | this work |
|  | 4.87 | 4.19 | 3.89 | 3.57 | ⎯ | 3.75 | 1.31 | ⎯ | 102.5 | 69.4 | 78.8 | 72.7 | 71.2 | 19.5 | ⎯ | Glushka ^e^ |
|  | 4.92 | 4.23 | 3.50 | 3.64 | ⎯ | 3.95 | 1.34 | ⎯ | 102.7 | 69.3 | 77.8 | 72.7 | 74.3 | 19.4 |  | Rodriguez^c^ |
| Aceric acid | 5.373 | 4.424 | ⎯ | 4.730 | ⎯ | 1.190 | ⎯ | ⎯ | 100.0 | 90.2 | 85.6 | 81.4 | 16.2 |  | ⎯ | this work |
|  | 5.38 | 4.39 | ⎯ | 4.84 | ⎯ | 1.19 | ⎯ | ⎯ | 100.4 | 90.4 |  | 80.5 | 15.3 | n.d. | ⎯ | Glushka ^e^ |
|  | 5.41 | 4.38 | ⎯ | 4.74 | ⎯ | 1.17 | ⎯ | ⎯ | 98.9 | 89.7 | 85.2 | 81.2 | 16.1 | n.d. |  | Rodriguez^c^ |
| Gal | 4.649 | 3.778 | 3.872 | 3.867 | ⎯ | 3.717 | 3.794 | ⎯ | 105.0 | 75.5 | 72.0 | 77.3 | 78.0 | 63.5 | ⎯ | this work |
| 2MFuc | 5.738 | 3.480 | 4.049 | 3.842 | ⎯ | 4.318 | 1.229 | 3.496 | 97.4 | 80.1 | 70.8 | 74.7 | 69.1 | 18.2 | 59.9 | this work |
|  | 5.65 | 3.45 | 4.00 | 3.81 | ⎯ | 4.37 | 1.17 | 3.47 | 97.8 | 80.1 | 70.9 | 74.6 | 69.0 | 18.1 | 60.0 | Glushka ^f^ |
|  | 5.732 | 3.48 | 4.10 | n.d. | ⎯ | 4.395 | 1.24 | 3.47 | 97.5 | 80.0 | 70.3 | n.d. | 68.8 | 18.1 |  | Rodriguez^c^ |

^a^ nomenclature of apiose: C3' is named in our table C5 and the corresponding protons H5 and H5'

^b^ Vidal et al. reported the chemical shifts of a 12-mer including a Gal-β1,2-Ace*f* (OAc)-β1,3-Rha-β1,3'-Api*f*-β1,2-Gal side chain; for better comparison 1.2 ppm was added to the reported ^13^C values and 0.023 ppm to the ^1^H chemical shifts

^c^ for better comparison reported ^13^C chemical shifts were corrected by + 2.3 ppm and ^1^H chemical shifts by + 0.12

^d^ Ishii et al. reported chemical shifts of free methyl apiofuranosides, to the reported ^13^C values 0.7 ppm was added, to ^1^H chemical shifts 0.1 ppm was added.

^e^ for better comparison 2 ppm was added to the reported ^13^C values.

^f^ for better comparison 0.2 ppm was added to the reported ^13^C values.

**Table S2** Observed chemical shifts of oligo after enzyme treatment, plus released monosaccharide referenced to DSS in comparison with related structures reported earlier (1-4).

| Moiety | H1 | H2 | H3 | H4 | H4' | H5 | H6/ H5' | H7 | C1 | C2 | C3 | C4 | C5 | C6 |  |
| --- | --- | --- | --- | --- | --- | --- | --- | --- | --- | --- | --- | --- | --- | --- | --- |
| β-Api ^a^ | 5.308 | 3.974 | ⎯ | 4.166 | 3.893 | 3.844 | n.d. | ⎯ | 104.6 | 80.1 | 80.4 | 75.8 | 72.0 | ⎯ | this work |
|  | 5.233 | 4.158 | ⎯ | 4.348 | 3.935 | 3.986 | 3.655 | ⎯ | 112.5 | n.d. | n.d. | 77.3 | 73.1 | ⎯ | Vidal ^b^ |
|  | 5.12 | n.d. | ⎯ | 4.23 | 3.81 | 4.08 | 3.53 | ⎯ | 112.5 | n.d. | n.d. | 76.9 | 73.3 | ⎯ | Rodriguez^c^ |
|  | 5.09 | 4.12 | ⎯ | 4.16 | 4.01 | 3.72 | 3.72 | ⎯ | 110.9 | 78.0 | 80.8 | 75.0 | 65.0 | ⎯ | Ishi ^d^ |
| α-Api ^a^ | 5.364 | 4.089 | ⎯ | 4.002 | 4.002 | n.d. | n.d. | ⎯ | 99.0 | 74.4 | 79.2 | 75.7 | n.d. | ⎯ | this work |
|  | 5.11 | 4.12 | ⎯ | 4.16 | 4.12 | 3.72 | 3.72 | ⎯ | 104.7 | 73.2 | 78.4 | 75.1 | 65.9 | ⎯ | Ishi ^d^ |
| Rha | 4.886 | 4.228 | 3.935 | 3.642 | ⎯ | 3.793 | 1.349 | ⎯ | 102.6 | 69.4 | 78.5 | 72.8 | 71.3 | 19.6 | this work |
|  | 4.87 | 4.19 | 3.89 | 3.57 | ⎯ | 3.75 | 1.31 | ⎯ | 102.5 | 69.4 | 78.8 | 72.7 | 71.2 | 19.5 | Glushka ^e^ |
|  | 4.92 | 4.23 | 3.50 | 3.64 | ⎯ | 3.95 | 1.34 | ⎯ | 102.7 | 69.3 | 77.8 | 72.7 | 74.3 | 19.4 | Rodriguez^c^ |
|  | 4.913 | 4.246 | 3.96 | 3.629 | ⎯ | 3.829 | 1.356 | ⎯ | 102.7 | 69.8 | n.d. | n.d. | n.d. | 19.7 | Vidal ^b^ |
| Aceric acid | 5.396 | 4.331 | ⎯ | 4.809 | ⎯ | 1.190 | ⎯ | ⎯ | 100.3 | 91.0 | 86.2 | 81.2 | 15.8 | ⎯ | this work |
|  | 5.38 | 4.39 | ⎯ | 4.84 | ⎯ | 1.19 | ⎯ | ⎯ | 100.4 | 90.4 |  | 80.5 | 15.3 | n.d. | Glushka ^e^ |
|  | 5.41 | 4.38 | ⎯ | 4.74 | ⎯ | 1.17 | ⎯ | ⎯ | 98.9 | 89.7 | 85.2 | 81.2 | 16.1 | n.d. | Rodriguez^c^ |
|  | 5.412 | 4.329 | ⎯ | 4.809 | ⎯ | 1.193 | ⎯ | ⎯ | 100.9 | 91.1 | n.d. | n.d. | 15.6 | n.d. | Vidal ^b^ |
| Gal (terminal) | 4.506 | 3.636 | 3.712 | 3.932 | ⎯ | 3.797 | 3.793 | ⎯ | 106.9 | 73.3 | 78.2 | 71.2 | 74.8 | 63.6 | this work |
|  | 4.503 | 3.633 | 3.669 | 3.934 | ⎯ | n.d. | 3.793 | ⎯ | 107.1 | 73.2 | 75.3 | 71.2 | n.d. | 63.7 | Vidal ^b^ |
| free β-2MeFuc | 4.608 | 3.187 | 3.687 | 3.754 | ⎯ | 3.790 | 1.254 | n.d. | 98.8 | 84.2 | 75.2 | 74.2 | 73.6 | 18.2 | this work |
| free α-2MeFuc | 5.454 | 3.479 | 3.896 | 3.812 | ⎯ | 4.193 | 1.220 | n.d. | 92.2 | 80.1 | 71.3 | 74.6 | 68.8 | 18.2 | this work |

^a^ nomenclature of apiose: C3' is named in our table C5 and the corresponding protons H5 and H5'

^b^ Vidal et al. reported the chemical shifts of a 12-mer including a Gal-β1,2-Ace*f* (OAc)-β1,3-Rha-β1,3'-Api*f*-β1,2-Gal side chain; for better comparison 1.2 ppm was added to the reported ^13^C values and 0.023 ppm to the ^1^H chemical shifts

^c^ for better comparison reported ^13^C chemical shifts were corrected by + 2.3 ppm and ^1^H chemical shifts by + 0.12

^d^ Ishii et al. reported chemical shifts of free methyl apiofuranosides, to the reported ^13^C values 0.7 ppm was added, to ^1^H chemical shifts 0.1 ppm was added.

**Table S3. X-ray data collection and refinement statistics**

Statistics for the highest-resolution shell are shown in parentheses

|  | **BT0984^GH139^** | **SDT091673 ^GH139^** |
| --- | --- | --- |
| **Data collection** |  |  |
| Beamline | iO4-1 (DLS) | i24 (DLS) |
| Wavelength (Å) | 0.98 | 0.999 |
| Resolution range (Å) | 47.43 – 2.70  (2.76 – 2.70) | 76.74 - 2.05  (2.123 - 2.05) |
| Space group | P2_1_2_1_2_1_ | P 6_5_22 |
| Unit cell  a, b, c (Å)  a, b, g (°) | 101.20, 138.22, 195.53  90, 90, 90 | 209.45 209.45 143.96  90.00 90.00 120.00 |
| Total reflections | 251340  (15118) | 4209044 (681959) |
| Unique reflections | 75053 (4450) | 5422 (4518) |
| Multiplicity | 3.3 (3.4) | 39.5 (24.4) |
| Completeness (%) | 98.9 (99.5) | 99.96 (99.92) |
| Mean I/sigma(I) | 10.4 (1.3) | 71.7 (4) |
| Wilson B-factor (Å^2^) | 59.22 | 26.62 |
| Rmerge | 0.089 (1.050) | 0.050 (0.773) |
| Rmeas | 0.105 (1.246) | 0.051 (0.789) |
| CC1/2 | 0.997 (0.502) | 1 (0.887) |
| **Refinement** |  |  |
| R-work/ R-free | 0.22/0.26 | 0.18/0.19 |
| RMS(bonds) (Å) | 0.005 | 0.01 |
| RMS(angles) (º) | 1.40 | 1.03 |
| Ramachandran favored (%) | 94.33 | 97.37 |
| Ramachandran allowed (%) | 99.93 | 99.87 |
| Ramachandran outliers (%) | 0.07 | 0.13 |
| Rotamer outliers (%) | 2.85 | 0.31 |
| Clashscore | 4.72 | 1.96 |
| Average B-factor (Å^2^) | 75.46 | 33.45 |
| macromolecules | 70.6/ 80.31 | 32.74 |
| ligands | 0 | 0 |
| solvent | 42.80 | 39.54 |
| PDB code | 9HYQ | 9HMB |

**Table S4 Melting temperatures of GH139 enzymes and their mutants.**

All melting temperatures were performed in 10 mM MOPS pH 7.0 with 150 mM NaCl deploying 5 μM protein unless stated.

| **Enzyme** | **Melting point** | **Confidence interval** |
| --- | --- | --- |
| **Run 1** | | |
| BT_0984_WT | 54.38 | 44.24 to 50.71 |
| W162A | 54.78 | 54.59 to 55.55 |
| W403A | 51.48 | 51.02 to 52.39 |
| Q411A | 55.01 | 54.03 to 56.05 |
| N412A | 54.22 | 53.61 to 55.23 |
| E472A | 56.56 | 55.78 to 57.29 |
| E472Q | 55.37 | 55.07 to 55.83 |
| W490A | 56.64 | 56.13 to 57.11 |
| E561A | 58.62 | 57.18 to 59.25 |
| E561Q | 57.21 | 55.22 to 60.26 |
| N639A | 56.07 | 55.54 to 56.99 |
| E641A | 54.33 | 53.10 to 55.66 |
| E641Q | 56.00 | 54.58 to 56.95 |
| W683A | 47.48 | 45.79 to 49.92 |
| **Run2** | | |
| SDT | 59.29 | 59.18 to 59.40 |
| SDT T115Q | 41.1 | 40.83 to 41.36 |
| Q411A + N412A | 53.96 | 53.45 to 54.81 |
| Q411A + E562Q | 58.91 | 59.17 to 59.74 |
| N412A + E562Q | 56.55 | 56.71 to 58.48 |
| Q411A + N412A + E562Q | 56.89 | 57.06 to 58.16 |

**Table S5. Total hydrogen bond interactions between 2-methoxy-α-fucose and BT0984 during molecular dynamics simulations.**

|  |  | **% of the simulation time** | |
| --- | --- | --- | --- |
| **Ligand Atom** | **AA residue** | **Simulation 1** | **Simulation 1** |
| Hemi acetal O | T409 | 11.30 | 3.00 |
| Hemi acetal O | Q411 | - | 3.30 |
| Hemi acetal O | N412 | 72.00 | 67.00 |
| Hemi acetal O | Q461 | 4.90 | 2.20 |
| Hemi acetal O | H2O | 74.80 | 96.10 |
| C1-O | Q461 | 1.60 | 1.90 |
| C1-O | Q461 | 25.80 | - |
| C1-O | H2O | 99.20 | 99.80 |
| C1-OH | E561 | 86.60 | 76.80 |
| C1-OH | E561 | 36.80 | 73.80 |
| C1-OH | H2O | 8.70 | 16.90 |
| C2-O | N639 | 1.40 | 14.40 |
| C2-O | H2O | 95.40 | 83.20 |
| C3-O | W683 | 93.30 | 90.20 |
| C3-O | Y722 | 6.00 | 5.60 |
| C3-O | H2O | 72.30 | 82.50 |
| C3-OH | H2O | 97.10 | 96.00 |
| C4-O | N412 | 99.90 | 98.30 |
| C4-O | W683 | 94.30 | 93.40 |
| C4-O | H2O | 13.60 | 11.30 |
| C4-OH | D723 | 83.40 | 100.00 |
| C4-OH | D723 | 94.80 | 77.30 |

**Table S6. Hydrogen bond occupancy at subsite −1 residues of BT0984 in complex with 2-methoxy-α-fucose.**

|  |  | **% of the simulation time** | |
| --- | --- | --- | --- |
| **AA residue** | **Ligand atom** | **Simulation 1** | **Simulation 2** |
| D723 | C4-OH | 83.40% | 100.00% |
| D723 | C4-OH | 94.80% | 77.30% |
| E561 | C1-OH | 86.60% | 76.81% |
| E561 | C1-OH | 36.80% | 73.80% |
| N412 | Hemi acetal O | 72.00% | 67.00% |
| N412 | C4-O | 99.90% | 98.30% |
| N729 | __ | 0.00% | 0.00% |
| Q411 | C2-O | 0% | 3.30% |
| W683 | C3-O | 93.30% | 90.20% |
| W683 | C4-O | 94.30% | 93.40% |

**Table S7. Binding free energy (ΔG_bind_) of 2-methoxy-α-fucose to BT0984 calculated by MM-GBSA (values in kcal/mol).**

|  | **Binding free energy** | **Van der Waal contribution** | **Electrostatic contribution** |
| --- | --- | --- | --- |
| **Simulation 1** | -18.05 | -19.13 | -43.80 |
| **Simulation 2** | -16.17 | -20.34 | -40.81 |

**Table S8. Mutant primer table**

| **Mutant primer** | **Primer sequence 5’ – 3’** |
| --- | --- |
| BT0984_W162A_F | TCC TAT AAG GCC GCTCCTCCAAAAGGAACGATGACG |
| BT0984_W162A_R | TGG AGG AGC GGC CTTATAGGAACATTGCTGCCCCTC |
| BT0984_W404A_F | TAC CGA AAG GCC GGAGGAGGAACGATGACTGCACAG |
| BT0984_W403A_R | GCA TTC ACT GGC GACTACCGAAAGTGGGGAGGAGGA |
| BT0984_Q461A_F | TTC TGT GAG GCC ATAGAAAACTTCGGCTTACCCAAT |
| BT0984_Q461A_R | GTT TTC TAT GGC CTCACAGAAGCAGGCTCCCTCGTG |
| BT0984_E472A_F | AAT CCC GCA GCC TATGGTTTCAAACGTCCGGCTTGG |
| BT0984_E472A_R | GAA ACC ATA GGC TGCGGGATTGGGTAAGCCGAAGTT |
| BT0984_E472Q_F | AAT CCC GCA CAG TATGGTTTCAAACGTCCGGCTTGG |
| BT0984_E472Q_R | GAA ACC ATA CTG TGCGGGATTGGGTAAGCCGAAGTT |
| BT0984_W490A_F | TAC AAT GCG GCC CTGGAATATGAATGGGATACTATT |
| BT0984_W490A_R | ATA TTC CAG GGC CGCATTGTATTCCAGTCCTTTGTC |
| BT0984_E561A_F | TCT GCC TGC GCC ACTTACAAGATGACCAACAACGCC |
| BT0984_E561A_R | CTT GTA AGT GGC GCAGGCAGAGCCGGGGAACAGTAT |
| BT0984_E561Q_F | TCT GCC TGC CAG ACTTACAAGATGACCAACAACGCC |
| BT0984_E561Q_R | CTT GTA AGT CTG GCAGGCAGAGCCGGGGAACAGTAT |
| BT0984_N639A_F | CGA ATC AAC GCC ATAGAGACACCACAACTCTACCCG |
| BT0984_N639A_R | TGT CTC TAT GGC GTTGATTCGTTCCCAGCTTTTGGC |
| BT0984_E641A_F | AAC AAT ATA GCC ACACCACAACTCTACCCGGTTTTT |
| BT0984_E641A_R | TTG TGG TGT GGC TATATTGTTGATTCGTTCCCAGCT |
| BT0984_E641Q_F | AAC AAT ATA CAG ACACCACAACTCTACCCGGTTTTT |
| BT0984_E641Q_R | TTG TGG TGT CTG TATATTGTTGATTCGTTCCCAGCT |
| BT0984_W683A_F | CAT ACC GGA GCC AAGCAAGACAACATCTGGGCAGCC |
| BT0984_W683A | GTC TTG CTT GGC TCCGGTATGGGAGCGGAATTTGAG |
| BT0984_N412A_F | ACT GCA CAG GCC CAGCGTCTTGTCTACTGGCCG |
| BT0984_N412A | AAG ACG CTG GGC CTGTGCAGTCATCGTTCCTCC |
| BT0984_Q411A_F | ATG ACT GCA GCC AATCAGCGTCTTGTCTACTGG |
| BT0984_Q411A_R | ACG CTG ATT GGC TGCAGTCATCGTTCCTCCTCC |
| BT0984_Q411A/N412A_F | ATG ACT GCA GCC GCC CAG CGT CTT GTC TAC TGG |
| BT0984_Q411A/N412A_R | ACG CTG GGC GGC TGC AGT CAT CGT TCC TCC TCC |
| SDT_T551E_F | CTGCTCAGAGCCTGGAGACCTACCAGTCCC |
| SDT_T551E_R | GGGACTGGTAGGTCTCCAGGCTCTGAGCAG |

1. Glushka, J. N., Terrell, M., York, W. S., O'Neill, M. A., Gucwa, A., Darvill, A. G. *et al.* (2003) Primary structure of the 2-O-methyl-alpha-L-fucose-containing side chain of the pectic polysaccharide, rhamnogalacturonan II Carbohydr Res **338**, 341-352 10.1016/s0008-6215(02)00461-5

2. Ishii, T., Yanagisawa, M (1998) Synthesis, separation and NMR spectral analysis of methyl apiofuranosides Carbohydrate Research **313**, 189-192 <https://doi.org/10.1016/S0008-6215(98)00262-6>

3. Rodriguez-Carvajal, M. A., Herve du Penhoat, C., Mazeau, K., Doco, T., andPerez, S. (2003) The three-dimensional structure of the mega-oligosaccharide rhamnogalacturonan II monomer: a combined molecular modeling and NMR investigation Carbohydr Res **338**, 651-671 10.1016/s0008-6215(03)00003-x

4. Vidal, S., Doco, T., Williams, P., Pellerin, P., York, W. S., O'Neill, M. A. *et al.* (2000) Structural characterization of the pectic polysaccharide rhamnogalacturonan II: evidence for the backbone location of the aceric acid-containing oligoglycosyl side chain Carbohydr Res **326**, 277-294 10.1016/s0008-6215(00)00036-7
